# Mass spectrometry based data of the blister fluid proteome of paediatric burn patients

**DOI:** 10.1016/j.dib.2016.07.033

**Published:** 2016-07-26

**Authors:** Tuo Zang, Daniel A. Broszczak, Leila Cuttle, James A. Broadbent, Catherine Tanzer, Tony J. Parker

**Affiliations:** a Tissue Repair and Regeneration Program, Institute of Health and Biomedical Innovation, Queensland University of Technology, Kelvin Grove, Queensland, Australia; b School of Biomedical Sciences, Faculty of Health, Queensland University of Technology, Brisbane, Queensland, Australia; c Wound Management Innovation Co-operative Research Centre, Brisbane, Queensland, Australia; d Centre for Children’s Burns and Trauma Research, Queensland University of Technology, Institute of Health and Biomedical Innovation at the Centre for Children’s Health Research, South Brisbane, Queensland, Australia

## Abstract

The data presented here are associated with the article “The blister fluid proteome of paediatric burns” (Zang et al., 2016) [1]. Burn injury is a highly traumatic event for children. The degree of burn severity (superficial-, deep-, or full-thickness injury) often dictates the extent of later scar formation which may require long term surgical operation or skin grafting. The data were obtained by fractionating paediatric burn blister fluid samples, which were pooled according to burn depth and then analysed using data dependent acquisition LC–MS/MS. The data includes a table of all proteins identified, in which burn depth category they were found, the percentage sequence coverage for each protein and the number of high confidence peptide identifications for each protein. Further Gene Ontology enrichment analysis shows the significantly over-represented biological processes, molecular functions, and cellular components of the burn blister fluid proteome. In addition, tables include the proteins associated with the biological processes of “wound healing” and “response to stress” as examples of highly relevant processes that occur in burn wounds.

**Specifications Table**

**Table:** 

Subject area	*Biochemistry*
More specific subject area	*Proteomics*
Type of data	*Table, Figures, and Cytoscape file*
How data was acquired	*LC-MS/MS, Eksigent ekspert 400 nanoLC system tandem TripleTOF 5600+ mass spectrometer (SCIEX)*
Data format	Raw, filtered and analysed
Experimental factors	*The blister fluid samples were pooled based on the depth classification, fractionated using 4 different methods, digested by trypsin and de-salted and enriched using Stage-Tips.*
Experimental features	*Data dependent acquisition LC- MS/MSGene ontology analysis*
Data source location	*Institute of Health and Biomedical Innovation (IHBI), Queensland University of Technology (QUT), Kelvin Grove, Queensland, Australia*
Data accessibility	*Data is provided with this article*

## **Value of the data**

• First and most comprehensive proteome of burn blister fluid that can be used to compare against other disease states/patient populations.
• These data provide a reference list of known, observed proteins within paediatric burn blister fluid, which will be of interest for the burn wound research community and clinicians.
• Qualitative evaluation of the biochemical differences between burns of different depths will enable future targeted quantitative analyses of protein abundance based on burn depth.
• The dataset allows for extensive Gene Ontology (GO) term analysis of the burn blister fluid proteome and the interaction/interrogation of this proteome through the Cytoscape data file provided herein.

## 1. Data

**Fig. 1:**
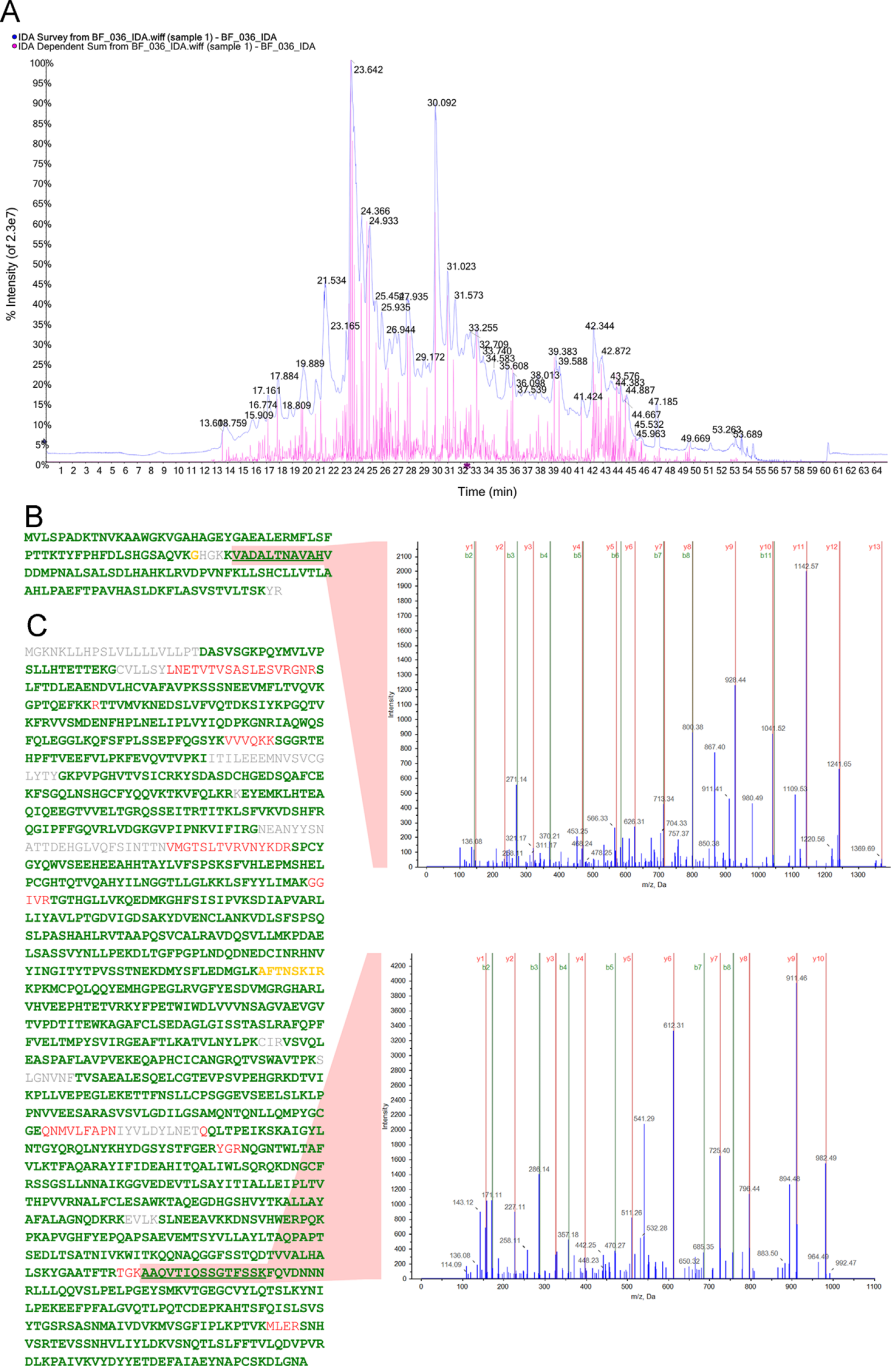
The mass spectra of an example sample. (A) Total ion chromatogram (blue) shows the complexity of ion information acquired from a single blister fluid sample. The dependent scan chromatogram (pink) shows the number of MS/MS ions detected. The total protein sequence coverage obtained and the corresponding MS2 spectra, with good *y*-ion series, of the underlined peptide are exemplified for burn relevant proteins, haemoglobin subunit alpha (B) and alpha-2 macroglobulin (C), respectively. Sequence coverage indicated by ≥95% confident peptides (green), ≥75% confident peptides (yellow), and ≥50% confident peptides (red). Grey amino acids were not detected.

**Fig. 2:**
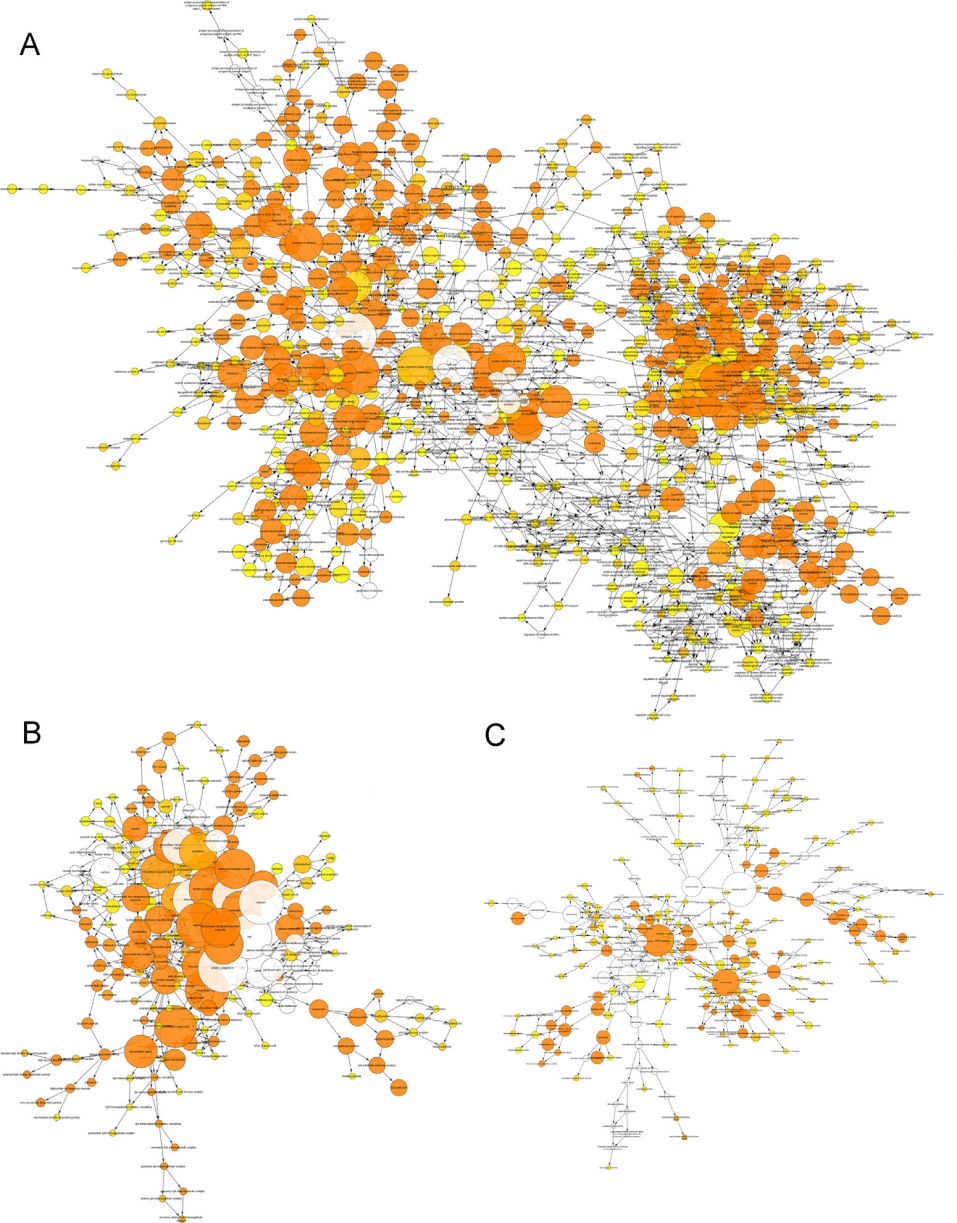
Gene ontology enrichment analysis for the entire protein inventory of burn blister fluid. The number of proteins involved in the annotation is in proportion to the size of nodes. The colour of the node represents the (corrected) *p*-value with a darker colour indicative of greater significance of over-representation for that GO term. Uncoloured nodes are the parents of over-represented downstream categories without over-representation themselves. (A) The network of biological process. (B) The network of cellular component. (C) The network of molecular function.

Presented in this publication is an inventory of proteins identified in paediatric burn blister fluid (1% FDR corrected), and the depths at which they were detected (Supplementary Table S1). For each protein, the following elements are provided: the UniProt accession number; description; detected presence in three different burn depths (superficial, deep partial and full thickness); ProteinPilot confidence score; percent sequence coverage; and the number of ≥95% confident peptides identified per protein. An example of the quality of the mass spectrometry data acquired is shown in Fig. 1. The Gene Ontology (GO) enrichment analysis of the whole protein library categorised by biological processes, molecular functions and cellular components in response to burn injury is shown in Fig. 2 and provided online as a Cytoscape file. The proteins specifically involved in the GO term biological process annotations for ‘*wound healing*’ and ‘*response to stress*’ are shown in Tables A1 and B1.

## 2. Experimental design, materials and methods

Methodology for blister fluid sample collection, sample preparation, liquid chromatography tandem mass spectrometry analysis, protein identification and GO analysis are described elsewhere [1].

### 2.1 Liquid chromatography tandem mass spectrometry (LC–MS/MS)

The quality of mass spectrometry data acquired and subsequently analysed was of a high standard (Fig. 1). The generation of complex and information rich ion chromatograms ensure that robust identifications of proteins are made (Fig. 1A). Furthermore, data acquired for proteins of relevance to burn injury were also of a high standard, with excellent sequence coverage and *y*-ion and *b*-ion series in MS2 spectra (Fig. 1B and C).

### 2.2 GO analysis

The over-represented biological processes (BP), molecular functions (MF), and cellular components (CC) were determined through Gene Ontology (GO) enrichment analysis of the whole blister fluid proteome using the BiNGO app within Cytoscape (Version 3.2.1, National Resource for Network Biology) (Fig. 2 and the Cytoscape file available online). Detected proteins within the two over-represented GO terms, ‘*wound healing*’ and ‘*response to stress*’ were compared across three burn depths (Tables A1 and B1, respectively). Subsets of these data with additional interpretation relevant to burn injury can be found elsewhere [1].

## Figures and Tables

**Table A1:** Distribution of significance level between burn severities for the biological process GO term, *wound healing*, and the burn depth that proteins associated with this GO term were detected. *S*=superficial thickness, *D*=deep partial thickness, *F*=full-thickness.

**Biological process of*****wound healing***				
**UniProt accession number**	**Cohort**	***S***	***D***	***F***
	**heatmap**			
	**corrected *p*-value**	1.55E−30	2.09E−35	3.11E−48
P11021	78 kDa glucose-regulated protein	X	X	✓
P60709	Actin, cytoplasmic 1	✓	✓	✓
P63261	Actin, cytoplasmic 2	✓	✓	✓
Q01518	Adenylyl cyclase-associated protein 1	X	X	✓
P01009	Alpha-1-antitrypsin	✓	✓	✓
P08697	Alpha-2-antiplasmin	✓	✓	✓
P01023	Alpha-2-macroglobulin	✓	✓	✓
P12814	Alpha-actinin-1	X	X	✓
O43707	Alpha-actinin-4	✓	✓	✓
P01008	Antithrombin-III	✓	✓	✓
P08519	Apolipoprotein	✓	✓	✓
P02647	Apolipoprotein A-I	✓	✓	✓
P04114	Apolipoprotein B-100	✓	✓	✓
P05090	Apolipoprotein D	✓	X	✓
P02749	Beta-2-glycoprotein 1	✓	✓	✓
P20851	C4b-binding protein beta chain	✓	X	X
Q96IY4	Carboxypeptidase B2	✓	✓	✓
P16070	CD44 antigen	✓	X	✓
O00299	Chloride intracellular channel protein 1	X	X	✓
P10909	Clusterin	✓	✓	✓
P00740	Coagulation factor IX	✓	✓	✓
A0A0A0MRJ7	Coagulation factor V	X	X	✓
P00742	Coagulation factor X	✓	✓	✓
P03951	Coagulation factor XI	✓	X	✓
P00748	Coagulation factor XII	✓	✓	✓
P00488	Coagulation factor XIII A chain	X	X	✓
P05160	Coagulation factor XIII B chain	✓	✓	✓
P02452	Collagen alpha-1	X	✓	✓
P02461	Collagen alpha-1	X	X	✓
P02741	C-reactive protein	✓	✓	✓
P07585	Decorin	X	X	✓
P15924	Desmoplakin	✓	✓	X
P02671	Fibrinogen alpha chain	✓	✓	✓
P02675	Fibrinogen beta chain	✓	✓	✓
P02679	Fibrinogen gamma chain	✓	✓	✓
P02751	Fibronectin	X	✓	✓
P23142	Fibulin-1	✓	✓	✓
P04075	Fructose-bisphosphate aldolase A	✓	X	✓
P06396	Gelsolin	✓	✓	✓
P04792	Heat shock protein beta-1	✓	✓	✓
P68871	Haemoglobin subunit beta	✓	✓	✓
P02042	Haemoglobin subunit delta	X	✓	✓
P69891	Haemoglobin subunit gamma-1	X	X	✓
P05546	Heparin cofactor 2	✓	✓	✓
P04196	Histidine-rich glycoprotein	✓	✓	✓
P68431	Histone H3.1	✓	X	✓
Q71DI3	Histone H3.2	X	X	✓
P03956	Interstitial collagenase	✓	✓	✓
Q92876	Kallikrein-6	X	✓	✓
P02538	Keratin, type II cytoskeletal 6A	X	✓	✓
P01042	Kininogen-1	✓	X	X
P14151	L-selectin	X	✓	✓
P01033	Metalloproteinase inhibitor 1	✓	✓	✓
P35579	Myosin-9	X	X	✓
P62937	Peptidyl-prolyl cis-trans isomerase A	✓	✓	✓
P03952	Plasma kallikrein	✓	✓	✓
P05155	Plasma protease C1 inhibitor	✓	✓	✓
P05154	Plasma serine protease inhibitor	✓	✓	X
P00747	Plasminogen	✓	✓	✓
P02775	Platelet basic protein	X	✓	✓
P07737	Profilin-1	✓	✓	✓
P05109	Protein S100-A8	✓	✓	✓
P00734	Prothrombin	✓	✓	✓
P02787	Serotransferrin	✓	✓	✓
P02768	Serum albumin	✓	✓	✓
P0DJI8	Serum amyloid A-1 protein	✓	✓	✓
P00441	Superoxide dismutase [Cu-Zn]	✓	✓	✓
P18827	Syndecan-1	X	X	✓
Q9Y490	Talin-1	X	X	✓
P07996	Thrombospondin-1	X	✓	✓
P18206	Vinculin	X	X	✓
P07225	Vitamin K-dependent protein S	✓	✓	✓
P04275	von Willebrand factor	X	✓	✓

**Table B1:** Distribution of significance level between burn severities for the biological process GO term, *response to stress*, and the burn depth that proteins associated with this GO term were detected. *S*=superficial thickness, *D*=deep partial thickness, *F*=full-thickness.

**Biological process of *r******esponse to stress***				
**UniProt accession number**	**Cohort**	***S***	***D***	***F***
	**heatmap**			
	**corrected *p*-value**	1.10E−61	1.01E−72	1.37E−76
P31946	14-3-3 protein beta/alpha	X	X	✓
P62258	14-3-3 protein epsilon	✓	X	X
P31947	14-3-3 protein sigma	✓	✓	✓
P11021	78 kDa glucose-regulated protein	X	X	✓
P60709	Actin, cytoplasmic 1	✓	✓	✓
P63261	Actin, cytoplasmic 2	✓	✓	✓
P61160	Actin-related protein 2	✓	X	X
O15145	Actin-related protein 2/3 complex subunit 3	✓	X	X
O15511	Actin-related protein 2/3 complex subunit 5	✓	✓	X
P61158	Actin-related protein 3	X	X	✓
Q01518	Adenylyl cyclase-associated protein 1	X	X	✓
Q15848	Adiponectin	✓	X	X
P02763	Alpha-1-acid glycoprotein 1	✓	✓	✓
P19652	Alpha-1-acid glycoprotein 2	✓	✓	✓
P01011	Alpha-1-antichymotrypsin	✓	✓	✓
P01009	Alpha-1-antitrypsin	✓	✓	✓
P08697	Alpha-2-antiplasmin	✓	✓	✓
P02765	Alpha-2-HS-glycoprotein	✓	✓	✓
P01023	Alpha-2-macroglobulin	✓	✓	✓
P12814	Alpha-actinin-1	X	X	✓
O43707	Alpha-actinin-4	✓	✓	✓
P01019	Angiotensinogen	✓	✓	✓
P04083	Annexin A1	✓	X	✓
P01008	Antithrombin-III	✓	✓	✓
P08519	Apolipoprotein	✓	✓	✓
P02647	Apolipoprotein A-I	✓	✓	✓
P06727	Apolipoprotein A-IV	✓	✓	✓
P04114	Apolipoprotein B-100	✓	✓	✓
P05090	Apolipoprotein D	✓	X	✓
P02649	Apolipoprotein E	✓	✓	✓
O14791	Apolipoprotein L1	✓	✓	✓
O75882	Attractin	✓	X	X
P02749	Beta-2-glycoprotein 1	✓	✓	✓
P04003	C4b-binding protein alpha chain	✓	✓	✓
P20851	C4b-binding protein beta chain	✓	X	X
P27797	Calreticulin	X	X	✓
P00918	Carbonic anhydrase 2	X	X	✓
Q96IY4	Carboxypeptidase B2	✓	✓	✓
P31944	Caspase-14	✓	X	✓
P04040	Catalase	X	X	✓
J3KNB4	Cathelicidin antimicrobial peptide	X	✓	X
P07858	Cathepsin B	X	X	✓
P16070	CD44 antigen	✓	X	✓
O43866	CD5 antigen-like	✓	✓	✓
O00299	Chloride intracellular channel protein 1	X	X	✓
P10909	Clusterin	✓	✓	✓
P00740	Coagulation factor IX	✓	✓	✓
A0A0A0MRJ7	Coagulation factor V	X	X	✓
P00742	Coagulation factor X	✓	✓	✓
P03951	Coagulation factor XI	✓	X	✓
P00748	Coagulation factor XII	✓	✓	✓
P00488	Coagulation factor XIII A chain	X	X	✓
P05160	Coagulation factor XIII B chain	✓	✓	✓
P02452	Collagen alpha-1	X	✓	✓
P02461	Collagen alpha-1	X	X	✓
P02745	Complement C1q subcomponent subunit A	✓	✓	✓
P02747	Complement C1q subcomponent subunit C	✓	✓	✓
Q9NZP8	Complement C1r subcomponent-like protein	✓	✓	✓
P09871	Complement C1s subcomponent	✓	✓	✓
P06681	Complement C2	✓	✓	✓
P01024	Complement C3	✓	✓	✓
P0C0L4	Complement C4-A	✓	✓	✓
P0C0L5	Complement C4-B	✓	✓	✓
P01031	Complement C5	✓	✓	✓
P13671	Complement component C6	✓	✓	✓
P10643	Complement component C7	✓	✓	✓
P07357	Complement component C8 alpha chain	✓	✓	✓
P07358	Complement component C8 beta chain	✓	✓	✓
P07360	Complement component C8 gamma chain	✓	✓	✓
P02748	Complement component C9	✓	✓	✓
P08603	Complement factor H	✓	✓	✓
P31146	Coronin-1A	✓	X	✓
P02741	C-reactive protein	✓	✓	✓
P01034	Cystatin-C	✓	✓	✓
P99999	Cytochrome c	✓	X	X
P07585	Decorin	X	X	✓
P81605	Dermcidin	✓	X	✓
P15924	Desmoplakin	✓	✓	X
P78527	DNA-dependent protein kinase catalytic subunit	✓	✓	X
Q16610	Extracellular matrix protein 1	✓	✓	✓
P08294	Extracellular superoxide dismutase [Cu–Zn]	✓	✓	✓
Q01469	Fatty acid-binding protein, epidermal	✓	✓	✓
P02671	Fibrinogen alpha chain	✓	✓	✓
P02675	Fibrinogen beta chain	✓	✓	✓
P02679	Fibrinogen gamma chain	✓	✓	✓
P02751	Fibronectin	X	✓	✓
P23142	Fibulin-1	✓	✓	✓
Q15485	Ficolin-2	X	X	✓
O75636	Ficolin-3	✓	✓	✓
P04075	Fructose-bisphosphate aldolase A	✓	X	✓
P17931	Galectin-3	✓	X	✓
Q08380	Galectin-3-binding protein	X	X	✓
P06396	Gelsolin	✓	✓	✓
A0A087X1J7	Glutathione peroxidase	✓	✓	✓
H0YBE4	Glutathione peroxidase 3	✓	X	X
P09211	Glutathione S-transferase P	✓	✓	✓
P04406	Glyceraldehyde-3-phosphate dehydrogenase	✓	✓	✓
P00738	Haptoglobin	✓	✓	✓
P00739	Haptoglobin-related protein	✓	✓	✓
P34931	Heat shock 70 kDa protein 1-like	X	✓	X
P11142	Heat shock cognate 71 kDa protein	✓	X	✓
P04792	Heat shock protein beta-1	✓	✓	✓
P08238	Heat shock protein HSP 90-beta	✓	✓	X
P69905	Haemoglobin subunit alpha	✓	✓	✓
P68871	Haemoglobin subunit beta	✓	✓	✓
P02042	Haemoglobin subunit delta	X	✓	✓
P69891	Haemoglobin subunit gamma-1	X	X	✓
P05546	Heparin cofactor 2	✓	✓	✓
P04196	Histidine-rich glycoprotein	✓	✓	✓
O60814	Histone H2B type 1-K	X	✓	X
P68431	Histone H3.1	✓	X	✓
Q71DI3	Histone H3.2	X	X	✓
Q9BYW2	Histone-lysine N-methyltransferase SETD2	X	✓	X
P01876	Ig alpha-1 chain C region	✓	✓	✓
P01877	Ig alpha-2 chain C region	X	X	✓
P01859	Ig gamma-2 chain C region	✓	✓	✓
P01861	Ig gamma-4 chain C region	✓	✓	✓
P01602	Ig heavy chain V-I region 5	✓	✓	✓
P01814	Ig heavy chain V-II region OU	✓	X	✓
P01764	Ig heavy chain V-III region 23	✓	X	✓
P01767	Ig heavy chain V-III region BUT	✓	X	X
P01781	Ig heavy chain V-III region GAL	✓	X	X
P01780	Ig heavy chain V-III region JON	X	✓	✓
P01762	Ig heavy chain V-III region TRO	✓	X	X
P01779	Ig heavy chain V-III region TUR	X	✓	✓
P01776	Ig heavy chain V-III region WAS	✓	X	X
P01593	Ig kappa chain V-I region AG	X	X	✓
P01613	Ig kappa chain V-I region Ni	X	✓	X
P01608	Ig kappa chain V-I region Roy	✓	X	X
P01609	Ig kappa chain V-I region Scw	X	✓	X
P06310	Ig kappa chain V-II region RPMI 6410	✓	X	X
P01617	Ig kappa chain V-II region TEW	✓	✓	✓
P18136	Ig kappa chain V-III region HIC	✓	✓	✓
P01621	Ig kappa chain V-III region NG9	X	X	✓
P06314	Ig kappa chain V-IV region B17	X	X	✓
P06313	Ig kappa chain V-IV region JI	✓	✓	✓
P06316	Ig lambda chain V-I region BL2	✓	X	X
P01701	Ig lambda chain V-I region NEW	X	X	✓
P04208	Ig lambda chain V-I region WAH	✓	X	✓
P80748	Ig lambda chain V-III region LOI	X	X	✓
P01714	Ig lambda chain V-III region SH	X	✓	✓
P0CG06	Ig lambda-3 chain C regions	✓	X	X
Q14624	Inter-alpha-trypsin inhibitor heavy chain H4	✓	✓	✓
P03956	Interstitial collagenase	✓	✓	✓
Q92876	Kallikrein-6	X	✓	✓
P08779	Keratin, type I cytoskeletal 16	✓	✓	✓
P04264	Keratin, type II cytoskeletal 1	✓	✓	✓
P02538	Keratin, type II cytoskeletal 6A	X	✓	✓
P01042	Kininogen-1	✓	X	X
E7ER44	Lactotransferrin	✓	✓	✓
E7EQB2	Lactotransferrin	X	✓	X
P09960	Leukotriene A-4 hydrolase	X	X	✓
P18428	Lipopolysaccharide-binding protein	✓	✓	✓
P14151	L-selectin	X	✓	✓
F8VV32	Lysozyme	X	X	✓
P61626	Lysozyme C	✓	✓	✓
P14174	Macrophage migration inhibitory factor	✓	✓	✓
P40925	Malate dehydrogenase, cytoplasmic	✓	✓	✓
P48740	Mannan-binding lectin serine protease 1	✓	✓	X
O00187	Mannan-binding lectin serine protease 2	X	X	✓
P11226	Mannose-binding protein C	✓	X	X
P01033	Metalloproteinase inhibitor 1	✓	✓	✓
P08571	Monocyte differentiation antigen CD14	✓	✓	✓
P05164	Myeloperoxidase	✓	✓	✓
P35579	Myosin-9	X	X	✓
Q96PD5	N-acetylmuramoyl-L-alanine amidase	✓	✓	✓
P59666	Neutrophil defensin 3	✓	✓	✓
P08246	Neutrophil elastase	✓	X	✓
P06748	Nucleophosmin	✓	✓	✓
O95497	Pantetheinase	X	✓	✓
P26022	Pentraxin-related protein PTX3	✓	X	X
P62937	Peptidyl-prolyl cis-trans isomerase A	✓	✓	✓
Q06830	Peroxiredoxin-1	✓	X	X
P32119	Peroxiredoxin-2	✓	✓	✓
P03952	Plasma kallikrein	✓	✓	✓
P05155	Plasma protease C1 inhibitor	✓	✓	✓
P05154	Plasma serine protease inhibitor	✓	✓	X
P00747	Plasminogen	✓	✓	✓
P02775	Platelet basic protein	X	✓	✓
P02545	Prelamin-A/C [Cleaved into: Lamin-A/C	✓	✓	✓
P07737	Profilin-1	✓	✓	✓
P25788	Proteasome subunit alpha type-3	✓	X	X
P28066	Proteasome subunit alpha type-5	✓	X	X
O14818	Proteasome subunit alpha type-7	X	X	✓
P28072	Proteasome subunit beta type-6	✓	✓	✓
P07237	Protein disulphide-isomerase	X	X	✓
P30101	Protein disulphide-isomerase A3	X	X	✓
P13667	Protein disulphide-isomerase A4	X	X	✓
Q92597	Protein NDRG1	X	X	✓
Q9HCY8	Protein S100-A14	✓	X	X
P31151	Protein S100-A7	X	X	✓
P05109	Protein S100-A8	✓	✓	✓
P06702	Protein S100-A9	✓	✓	✓
A0A096LPE2	Protein SAA2-SAA4	✓	✓	✓
P00734	Prothrombin	✓	✓	✓
P55786	Puromycin-sensitive aminopeptidase	X	X	✓
P02787	Serotransferrin	✓	✓	✓
P02768	Serum albumin	✓	✓	✓
E9PQD6	Serum amyloid A protein	X	✓	X
P0DJI8	Serum amyloid A-1 protein	✓	✓	✓
P0DJI9	Serum amyloid A-2 protein	X	✓	✓
P02743	Serum amyloid P-component	✓	✓	✓
P08254	Stromelysin-1	X	X	✓
P00441	Superoxide dismutase [Cu-Zn]	✓	✓	✓
P18827	Syndecan-1	X	X	✓
Q9Y490	Talin-1	X	X	✓
P10599	Thioredoxin	✓	✓	✓
P07996	Thrombospondin-1	X	✓	✓
P55072	Transitional endoplasmic reticulum ATPase	✓	✓	✓
P35030	Trypsin-3	X	X	✓
P07437	Tubulin beta chain	✓	✓	X
P68371	Tubulin beta-4B chain	✓	X	X
P62987	Ubiquitin-60S ribosomal protein L40	✓	✓	✓
P18206	Vinculin	X	X	✓
P07225	Vitamin K-dependent protein S	✓	✓	✓
P04004	Vitronectin	✓	✓	✓
P04275	von Willebrand factor	X	✓	✓

## Appendix

See Table A1, Table B1
