# Supplementary material for: Mass spectrometry based data of the blister fluid proteome of paediatric burn patients
Source: Data Brief. 2016 Jul 26;8:1099–110. doi: 10.1016/j.dib.2016.07.033 (PMC4976667; doi:10.1016/j.dib.2016.07.033)
Supplement: Supplementary file 1 — Supplementary material [file mmc1.pdf]

QUT ihbi
Institute of Health and Biomedical Innovation

Queensland University of Technology

60 Musk Avenue Kelvin Grove Qld 4059 Australia
Phone 07 3138 6000 **www.ihbi.qut.edu.au**

CRICOS No. 00213J ABN 83 791 724 622

Conflict of Interest Statement

Manuscript No.: DIB-D-16-00514

New title: Mass spectrometry based data of the blister fluid proteome of paediatric burn patients

Journal Title: Data in Brief

Authors: Tuo Zang[1,2,3], Daniel A. Broszczak[1,2,3], Leila Cuttle[1,2,4], James A. Broadbent[1,2,3], Catherine Tanzer[1,3,4], Tony J. Parker[1,2]

Affiliations:

1. Tissue Repair and Regeneration Program, Institute of Health and Biomedical Innovation, Queensland University of Technology, Kelvin Grove, Queensland, Australia.
2. School of Biomedical Sciences, Faculty of Health, Queensland University of Technology, Brisbane, Queensland, Australia.
3. Wound Management Innovation Co-operative Research Centre, Brisbane, Queensland, Australia.
4. Centre for Children's Burns and Trauma Research, Queensland University of Technology, Institute of Health and Biomedical Innovation at the Centre for Children's Health Research, South Brisbane, Queensland, Australia.

Contact email: a.parker@qut.edu.au

On behalf of the authors, I would like to state that:

We certify that all the authors have no conflicts of interest. The authors have no other relevant affiliations or financial involvement with any organisation or entity with a financial interest in or financial conflict with the subject matter or materials discussed in the manuscript apart from those disclosed. This includes employment, consultancies, honoraria, stock ownership or options, expert testimony, grants or patents received or pending, or royalties.

Sincerely,

Dr Tony Parker, PhD
