# Supplementary material for: Mass spectrometry based data of the blister fluid proteome of paediatric burn patients
Source: Data Brief. 2016 Jul 26;8:1099–110. doi: 10.1016/j.dib.2016.07.033 (PMC4976667; doi:10.1016/j.dib.2016.07.033)
Supplement: Supplementary file 5 — Supplementary material [file mmc5.pdf]

Data in Brief article

Supplementary Data

Title: Mass spectrometry based data of the blister fluid proteome of paediatric burn patients

Authors: Tuo Zang[1,2,3], Daniel A. Broszczak[1,2,3], Leila Cuttle[1,2,4], James A. Broadbent[1,2,3], Catherine Tanzer[1,3,4], Tony J. Parker[1,2]

Affiliations:

1. Tissue Repair and Regeneration Program, Institute of Health and Biomedical Innovation, Queensland University of Technology, Kelvin Grove, Queensland, Australia.
2. School of Biomedical Sciences, Faculty of Health, Queensland University of Technology, Brisbane, Queensland, Australia.
3. Wound Management Innovation Co-operative Research Centre, Brisbane, Queensland, Australia.
4. Centre for Children's Burns and Trauma Research, Queensland University of Technology, Institute of Health and Biomedical Innovation at the Centre for Children's Health Research, South Brisbane, Queensland, Australia.

Contact email: a.parker@qut.edu.au

Supplementary Table S1. The entire protein inventory identified in the blister fluid from burn injuries

Accession number	Name	S	D	F	Unused Score	Coverage (%)	95% confident peptide
**sw|P61604**	10 kDa heat shock protein, mitochondrial	✓	✓	✓	5.47	31.37	3
**sw|P31946**	14-3-3 protein beta/alpha	✓	✓	✓	8.61	47.97	13
**sw|P62258**	14-3-3 protein epsilon	✓	✓	✓	6.06	28.63	8
**sw|Q04917**	14-3-3 protein eta	X	X	✓	2.54	19.11	7
**sw|P61981**	14-3-3 protein gamma	✓	✓	✓	6.55	27.13	11
**sw|P31947**	14-3-3 protein sigma	✓	✓	✓	38.19	74.6	29
**sw|P27348**	14-3-3 protein theta	X	X	✓	1.42	24.9	9
**tr|E7EX29**	14-3-3 protein zeta/delta (Fragment)	✓	✓	✓	19.97	62.6	19
**sw|Q99943**	1-acyl-sn-glycerol-3-phosphate acyltransferase alpha	✓	X	X	0.67	3.53	1
**sw|Q99714**	3-hydroxyacyl-CoA dehydrogenase type-2	✓	X	X	1.72	4.6	1
**sw|P25398**	40S ribosomal protein S12	✓	X	X	3.39	14.39	3
**tr|H0YB22**	40S ribosomal protein S14 (Fragment)	✓	X	X	2	9.17	1
**tr|M0R0R2**	40S ribosomal protein S5	✓	X	X	1.7	6.67	1
**tr|A0A0C4DG17**	40S ribosomal protein SA	✓	✓	X	3.04	7	2
**tr|C9J9K3**	40S ribosomal protein SA (Fragment)	✓	X	X	4	7.99	2

Accession number	Name	S	D	F	Unused Score	Coverage (%)	95% confident peptide
**tr|M0R1K5**	5-methylcytosine rRNA methyltransferase NSUN4	X	✓	X	2	5.3	1
**tr|F8VWS0**	60S acidic ribosomal protein P0	✓	X	X	7.26	26.33	4
**tr|F8VU65**	60S acidic ribosomal protein P0 (Fragment)	X	✓	✓	1.7	17.41	3
**sw|P05386**	60S acidic ribosomal protein P1	✓	✓	X	2	14.04	1
**sw|P05387**	60S acidic ribosomal protein P2	✓	✓	✓	4.01	24.35	2
**tr|H0YDD8**	60S acidic ribosomal protein P2 (Fragment)	✓	X	X	2	13.04	1
**sw|P30050**	60S ribosomal protein L12	✓	✓	X	2	5.45	1
**tr|K7ERI7**	60S ribosomal protein L22	✓	X	X	3.42	25.26	2
**tr|E5RJH3**	60S ribosomal protein L30	✓	X	X	2	21.43	1
**sw|P52209**	6-phosphogluconate dehydrogenase, decarboxylating	X	X	✓	6.17	10.97	4
**sw|P11021**	78 kDa glucose-regulated protein	✓	✓	✓	16.18	22.48	10
**sw|P68133**	Actin, alpha skeletal muscle	X	X	✓	38.65	41.91	30
**sw|P60709**	Actin, cytoplasmic 1	✓	✓	✓	35.76	76	58
**sw|P63261**	Actin, cytoplasmic 2	✓	✓	✓	57.91	76	56
**sw|P61160**	Actin-related protein 2	✓	X	X	2	3.05	1
**sw|Q92747**	Actin-related protein 2/3 complex subunit 1A	✓	✓	✓	2.13	4.87	2
**sw|O15143**	Actin-related protein 2/3 complex subunit 1B	✓	X	✓	2.89	7.53	2

Accession number	Name	S	D	F	Unused Score	Coverage (%)	95% confident peptide
**sw|O15145**	Actin-related protein 2/3 complex subunit 3	✓	X	✓	4.04	19.66	3
**tr|F8VR50**	Actin-related protein 2/3 complex subunit 3 (Fragment)	X	✓	✓	2	13.1	1
**sw|O15511**	Actin-related protein 2/3 complex subunit 5	✓	✓	✓	2.02	7.95	1
**sw|P61158**	Actin-related protein 3	X	X	✓	2.15	7.42	2
**sw|P53999**	Activated RNA polymerase II transcriptional coactivator p15	✓	X	X	0.95	4.72	1
**tr|Q5T0R9**	Adenylyl cyclase-associated protein (Fragment)	X	X	✓	2	4.96	1
**sw|Q01518**	Adenylyl cyclase-associated protein 1	✓	✓	✓	3.96	7.58	2
**sw|Q15848**	Adiponectin	✓	✓	✓	2.86	12.3	2
**tr|C9J1Z8**	ADP-ribosylation factor 5 (Fragment)	✓	X	X	1.3	7.33	1
**sw|P43652**	Afamin	✓	✓	✓	59.37	55.76	40
**sw|Q5JQC9**	A-kinase anchor protein 4	X	✓	X	0.23	1.64	1
**sw|P11766**	Alcohol dehydrogenase class-3	X	X	✓	2	2.14	1
**tr|J3KPS3**	Aldolase A, fructose-bisphosphate, isoform CRA_b	✓	✓	✓	24.6	41.3	20
**sw|P02763**	Alpha-1-acid glycoprotein 1	✓	✓	✓	87.67	75.62	138
**sw|P19652**	Alpha-1-acid glycoprotein 2	✓	✓	✓	25.39	74.13	74
**sw|P01011**	Alpha-1-antichymotrypsin	✓	✓	✓	114.19	72.58	132
**sw|P01009**	Alpha-1-antitrypsin	✓	✓	✓	134.86	81.82	311

Accession number	Name	S	D	F	Unused Score	Coverage (%)	95% confident peptide
**sw|P04217**	Alpha-1B-glycoprotein	✓	✓	✓	92.11	70.71	102
**sw|P08697**	Alpha-2-antiplasmin	✓	✓	✓	39.08	56.01	35
**sw|P02765**	Alpha-2-HS-glycoprotein	✓	✓	✓	74.53	77.11	141
**sw|P01023**	Alpha-2-macroglobulin	✓	✓	✓	450.74	82.77	618
**sw|P12814**	Alpha-actinin-1	X	X	✓	2.07	16.03	11
**sw|O43707**	Alpha-actinin-4	✓	✓	✓	34.19	29.75	19
**sw|P06733**	Alpha-enolase	✓	✓	✓	28.57	54.61	19
**sw|P03950**	Angiogenin	X	X	✓	1.96	10.88	1
**sw|P01019**	Angiotensinogen	✓	✓	✓	58.52	53.2	52
**tr|Q5T3N1**	Annexin (Fragment)	X	X	✓	1.08	22.55	3
**sw|P04083**	Annexin A1	✓	X	✓	3.14	13.29	3
**sw|P07355**	Annexin A2	X	X	✓	9.76	36.58	10
**tr|D6RA82**	Annexin A3	X	X	✓	2	3.17	1
**sw|P03973**	Antileukoproteinase	✓	X	✓	4.88	17.42	3
**sw|P01008**	Antithrombin-III	✓	✓	✓	87.02	65.73	84
**sw|P02647**	Apolipoprotein A-I	✓	✓	✓	235.8	93.26	379
**tr|V9GYM3**	Apolipoprotein A-II	✓	✓	✓	52.17	60.9	83

Accession number	Name	S	D	F	Unused Score	Coverage (%)	95% confident peptide
**sw|P06727**	Apolipoprotein A-IV	✓	✓	✓	158.82	83.08	134
**sw|P04114**	Apolipoprotein B-100	✓	✓	✓	369.96	47.51	293
**sw|P02654**	Apolipoprotein C-I	✓	✓	✓	19.29	49.4	14
**tr|K7ERI9**	Apolipoprotein C-I (Fragment)	✓	✓	✓	13.09	53.25	14
**tr|B0YIW2**	Apolipoprotein C-III	✓	✓	✓	14.08	49.57	10
**sw|P05090**	Apolipoprotein D	✓	✓	✓	26.21	44.97	26
**tr|C9JF17**	Apolipoprotein D (Fragment)	✓	✓	✓	26.62	38.6	21
**sw|P02649**	Apolipoprotein E	✓	✓	✓	46.16	72.56	35
**sw|Q13790**	Apolipoprotein F	✓	✓	✓	5.39	11.66	3
**sw|O14791**	Apolipoprotein L1	✓	✓	✓	12.92	20.6	7
**sw|O95445**	Apolipoprotein M	✓	✓	✓	14.75	51.6	8
**sw|P08519**	Apolipoprotein(a)	✓	✓	✓	6.76	27.22	15
**tr|E7EQT4**	Apoptotic chromatin condensation inducer in the nucleus	X	✓	✓	1.73	0.61	1
**sw|Q8WZ64**	Arf-GAP with Rho-GAP domain, ANK repeat and PH domain-containing protein 2	X	✓	✓	1.13	1.11	2
**sw|P05089**	Arginase-1	✓	X	X	2	3.42	1
**sw|Q5FYA8**	Arylsulfatase H	X	✓	X	0.5	1.25	1
**sw|P00505**	Aspartate aminotransferase, mitochondrial	X	X	✓	4	3.95	2

Accession number	Name	S	D	F	Unused Score	Coverage (%)	95% confident peptide
**sw|P25705**	ATP synthase subunit alpha, mitochondrial	✓	X	X	2	2.89	2
**sw|Q9ULI0**	ATPase family AAA domain-containing protein 2B	X	✓	X	2	0.55	1
**sw|O75882**	Attractin	✓	✓	✓	10.63	4.41	7
**sw|P20160**	Azurocidin	✓	X	✓	6.43	13.55	3
**sw|P02749**	Beta-2-glycoprotein 1	✓	✓	✓	81.1	70.14	90
**tr|F5H6I0**	Beta-2-microglobulin	✓	✓	✓	6.03	22.77	4
**sw|Q96KN2**	Beta-Ala-His dipeptidase	✓	✓	✓	1.41	8.28	3
**sw|P43251**	Biotinidase	X	✓	X	3.19	13.81	6
**sw|P07738**	Bisphosphoglycerate mutase	X	X	✓	1.06	4.63	1
**tr|K7ESE8**	Bleomycin hydrolase (Fragment)	✓	X	X	2	4.76	1
**sw|P80723**	Brain acid soluble protein 1	X	✓	✓	8	32.16	5
**sw|P04003**	C4b-binding protein alpha chain	✓	✓	✓	47.53	56.95	32
**sw|P20851**	C4b-binding protein beta chain	✓	✓	✓	12	26.19	8
**tr|A0A087WXI5**	Cadherin-1	✓	X	✓	7.83	5.09	4
**sw|P33151**	Cadherin-5	✓	✓	✓	14.6	12.12	9
**sw|Q9NP80**	Calcium-independent phospholipase A2-gamma	X	X	✓	1.26	1.28	1
**tr|H0Y7A7**	Calmodulin (Fragment)	✓	✓	✓	17.58	58.29	18

Accession number	Name	S	D	F	Unused Score	Coverage (%)	95% confident peptide
**sw|P27482**	Calmodulin-like protein 3	✓	✓	✓	10.01	66.44	13
**sw|Q9NZT1**	Calmodulin-like protein 5	✓	✓	✓	28.07	76.71	42
**sw|Q5T5Y3**	Calmodulin-regulated spectrin-associated protein 1	X	✓	X	2	0.37	1
**tr|A0A0C4DGB5**	Calpastatin	X	✓	X	10	10.61	5
**sw|P27797**	Calreticulin	X	X	✓	5.46	13.67	3
**sw|Q8NET6**	Carbohydrate sulfotransferase 13	✓	X	X	0.83	4.11	1
**sw|P00915**	Carbonic anhydrase 1	X	X	✓	22.08	57.47	21
**tr|E5RHP7**	Carbonic anhydrase 1 (Fragment)	✓	X	✓	14.94	49.8	20
**sw|P00918**	Carbonic anhydrase 2	X	X	✓	7.85	21.15	5
**sw|Q96IY4**	Carboxypeptidase B2	✓	✓	✓	9.21	16.08	5
**sw|O75976**	Carboxypeptidase D	X	✓	X	2	0.87	1
**sw|P15169**	Carboxypeptidase N catalytic chain	✓	✓	✓	13.01	24.89	9
**sw|P22792**	Carboxypeptidase N subunit 2	✓	✓	✓	20.2	30.28	13
**tr|A0A0C4DFP6**	Cartilage acidic protein 1	✓	X	X	5.86	5.49	4
**tr|G3XAP6**	Cartilage oligomeric matrix protein	✓	✓	✓	22.22	26.8	12
**sw|P31944**	Caspase-14	✓	✓	✓	10.02	35.95	7

Accession number	Name	S	D	F	Unused Score	Coverage (%)	95% confident peptide
**sw|P04040**	Catalase	X	✓	✓	15.9	25.05	10
**tr|B4DGU4**	Catenin beta-1	X	X	✓	0.76	1.55	1
**tr|J3KNB4**	Cathelicidin antimicrobial peptide	X	✓	X	2	4.62	1
**sw|P07858**	Cathepsin B	X	X	✓	2	5.31	1
**sw|P07339**	Cathepsin D	X	X	✓	5.94	14.81	4
**sw|O60911**	Cathepsin L2	X	X	✓	2.01	3.59	1
**sw|P16070**	CD44 antigen	✓	✓	✓	6	4.18	3
**tr|H0YDW7**	CD44 antigen (Fragment)	X	X	✓	2	12.92	3
**sw|O43866**	CD5 antigen-like	✓	✓	✓	22.97	34.01	14
**sw|P29373**	Cellular retinoic acid-binding protein 2	X	X	✓	2	6.52	1
**sw|P00450**	Ceruloplasmin	✓	✓	✓	194.07	74.08	224
**sw|O00299**	Chloride intracellular channel protein 1	X	X	✓	1.36	4.98	1
**sw|P06276**	Cholinesterase	✓	X	X	5.85	7.97	5
**sw|Q9P2M7**	Cingulin	X	X	✓	2.01	0.5	1
**sw|P10909**	Clusterin	✓	✓	✓	75.15	55.9	94
**sw|P00740**	Coagulation factor IX	✓	✓	✓	5.47	9.54	4
**tr|A0A0A0MRJ7**	Coagulation factor V	X	✓	✓	2.2	1.57	5

Accession number	Name	S	D	F	Unused Score	Coverage (%)	95% confident peptide
**sw|P00742**	Coagulation factor X	✓	✓	✓	4.69	6.97	3
**sw|P03951**	Coagulation factor XI	✓	✓	✓	13.58	15.36	11
**sw|P00748**	Coagulation factor XII	✓	✓	✓	27.27	38.21	23
**sw|P00488**	Coagulation factor XIII A chain	X	X	✓	0.79	2.19	1
**sw|P05160**	Coagulation factor XIII B chain	✓	✓	✓	8.67	12.86	6
**tr|E9PK25**	Cofilin-1	✓	✓	✓	10.18	30.39	5
**tr|E9PKB1**	Coiled-coil domain-containing protein 15	✓	X	X	0.7	1.46	1
**sw|P02452**	Collagen alpha-1(I) chain	✓	✓	✓	78.05	43.17	74
**sw|P02461**	Collagen alpha-1(III) chain	✓	✓	✓	22.82	18.01	20
**tr|A0A087X0S5**	Collagen alpha-1(VI) chain	X	X	✓	2.28	1.85	1
**sw|Q9UMD9**	Collagen alpha-1(XVII) chain	X	X	✓	0.87	0.87	1
**tr|A0A087WTA8**	Collagen alpha-2(I) chain	✓	✓	✓	57.11	36.88	45
**tr|E7ENL6**	Collagen alpha-3(VI) chain	X	X	✓	7.02	2.88	5
**sw|P02745**	Complement C1q subcomponent subunit A	✓	✓	✓	11.59	27.35	7
**tr|X6RLJ0**	Complement C1q subcomponent subunit A (Fragment)	X	✓	✓	6	15.91	6
**tr|D6R934**	Complement C1q subcomponent subunit B	✓	✓	✓	12.68	26.69	9
**sw|P02747**	Complement C1q subcomponent subunit C	✓	✓	✓	8.48	25.31	7

Accession number	Name	S	D	F	Unused Score	Coverage (%)	95% confident peptide
**tr|B4DPQ0**	Complement C1r subcomponent	✓	✓	✓	48.23	47.43	29
**sw|Q9NZP8**	Complement C1r subcomponent-like protein	✓	✓	✓	9.47	23.82	10
**sw|P09871**	Complement C1s subcomponent	✓	✓	✓	50.93	53.34	32
**sw|P06681**	Complement C2	✓	✓	✓	9.85	31.91	23
**sw|P01024**	Complement C3	✓	✓	✓	475.71	87.91	595
**sw|P0C0L4**	Complement C4-A	✓	✓	✓	206.44	71.16	196
**sw|P0C0L5**	Complement C4-B	✓	✓	✓	225.45	70.7	195
**sw|P01031**	Complement C5	✓	✓	✓	105.27	43.2	65
**sw|P13671**	Complement component C6	✓	✓	✓	52.02	40.36	30
**sw|P10643**	Complement component C7	✓	✓	✓	50.4	48.16	31
**sw|P07357**	Complement component C8 alpha chain	✓	✓	✓	31	41.44	21
**sw|P07358**	Complement component C8 beta chain	✓	✓	✓	49.76	53.3	29
**sw|P07360**	Complement component C8 gamma chain	✓	✓	✓	17.45	53.96	10
**sw|P07360**	Complement component C8 gamma chain (Fragment)	X	✓	X	12	40.1	8
**sw|P02748**	Complement component C9	✓	✓	✓	53.83	55.64	45
**tr|K7ERG9**	Complement factor D	✓	✓	✓	10.05	27.31	5
**sw|P08603**	Complement factor H	✓	✓	✓	177.57	68.56	157

Accession number	Name	S	D	F	Unused Score	Coverage (%)	95% confident peptide
**sw|Q03591**	Complement factor H-related protein 1	✓	✓	✓	4.61	59.09	28
**sw|P36980**	Complement factor H-related protein 2	✓	✓	✓	15.2	44.81	13
**sw|Q92496**	Complement factor H-related protein 4	✓	X	X	3.21	5.02	2
**tr|G3XAM2**	Complement factor I	✓	✓	✓	50.04	43.06	33
**tr|G8JLG2**	Corneodesmosin	✓	X	X	1.14	3.4	2
**sw|P35321**	Cornifin-A	✓	X	X	1.14	17.98	1
**sw|P31146**	Coronin-1A	✓	✓	✓	2.98	4.34	2
**sw|P08185**	Corticosteroid-binding globulin	✓	✓	✓	16	29.14	14
**sw|P02741**	C-reactive protein	✓	✓	✓	16.98	29.02	15
**sw|P12532**	Creatine kinase U-type, mitochondrial	✓	✓	✓	3.01	4.08	2
**sw|P01040**	Cystatin-A	✓	✓	X	6.1	39.8	3
**sw|P04080**	Cystatin-B	X	X	✓	4	24.49	2
**sw|P01034**	Cystatin-C	✓	✓	✓	8	31.51	6
**tr|E9PS42**	Cysteine and glycine-rich protein 1	X	X	✓	2.01	11.11	1
**tr|J3KPA1**	Cysteine-rich secretory protein 3	✓	✓	✓	2.05	3.26	1
**sw|P99999**	Cytochrome c	✓	X	X	2.01	20.95	2
**tr|C9JFR7**	Cytochrome c (Fragment)	✓	✓	✓	4.03	21.78	2

Accession number	Name	S	D	F	Unused Score	Coverage (%)	95% confident peptide
**sw|O43175**	D-3-phosphoglycerate dehydrogenase	✓	X	X	4.73	10.32	5
**tr|J3KQ18**	D-dopachrome decarboxylase	X	X	✓	2.08	9.09	1
**sw|P07585**	Decorin	X	X	✓	1.6	3.62	1
**sw|P13716**	Delta-aminolevulinic acid dehydratase	✓	X	X	2	3.03	1
**sw|P81605**	Dermcidin	✓	✓	✓	4	20	2
**sw|Q6E0U4**	Dermokine	X	X	✓	0.93	5.67	3
**sw|Q08554**	Desmocollin-1	✓	✓	X	4.89	3.69	3
**sw|Q14574**	Desmocollin-3	✓	X	X	1.59	4.13	3
**sw|Q02413**	Desmoglein-1	✓	X	✓	1.8	1.33	1
**sw|Q14126**	Desmoglein-2	X	✓	X	4	1.79	2
**sw|P15924**	Desmoplakin	✓	✓	X	4.54	1.74	6
**tr|I3L112**	Diacylglycerol kinase (Fragment)	X	X	✓	2	1.32	1
**tr|A0A087WZ02**	Diacylglycerol kinase eta (Fragment)	X	X	✓	1.43	11.29	1
**sw|Q9BPU6**	Dihydropyrimidinase-related protein 5	✓	X	X	0.51	1.24	1
**sw|P27487**	Dipeptidyl peptidase 4	✓	X	X	1.19	1.31	1
**sw|P78527**	DNA-dependent protein kinase catalytic subunit	✓	✓	X	2	0.31	2
**sw|Q9Y5R5**	Doublesex- and mab-3-related transcription factor 2	X	X	✓	0.95	1.96	1

Accession number	Name	S	D	F	Unused Score	Coverage (%)	95% confident peptide
**sw|Q5VZB9**	Doublesex- and mab-3-related transcription factor A1	X	✓	X	0.86	1.39	1
**tr|E7EN20**	E3 ubiquitin-protein ligase TRIM33	✓	✓	X	2	0.8	1
**tr|H0Y612**	E3 ubiquitin-protein ligase TRIM33 (Fragment)	X	X	✓	2	1.01	1
**tr|H0Y3M3**	Echinoderm microtubule-associated protein-like 3 (Fragment)	✓	X	X	0.89	1.21	3
**sw|Q12805**	EGF-containing fibulin-like extracellular matrix protein 1	X	X	✓	11.79	17.65	7
**sw|P19957**	Elafin	✓	✓	✓	16	52.14	10
**sw|P68104**	Elongation factor 1-alpha 1	X	X	✓	2.03	28.57	14
**sw|P13639**	Elongation factor 2	✓	X	✓	18.09	15.73	10
**sw|P49411**	Elongation factor Tu, mitochondrial	X	X	✓	1.8	1.55	1
**sw|Q9UNN8**	Endothelial protein C receptor	X	✓	✓	2	3.78	1
**tr|E7EMS2**	Epididymal secretory protein E1	X	X	✓	2.63	19.4	2
**tr|E7EQG2**	Eukaryotic initiation factor 4A-II	✓	X	X	2	2.76	1
**sw|Q13347**	Eukaryotic translation initiation factor 3 subunit I	✓	X	X	0.66	2.77	1
**tr|F8WCJ1**	Eukaryotic translation initiation factor 5A	✓	✓	✓	1.28	11.43	1
**sw|Q16610**	Extracellular matrix protein 1	✓	✓	✓	8.74	16.3	7
**sw|P08294**	Extracellular superoxide dismutase [Cu-Zn]	✓	✓	✓	4	10	2
**tr|E7EQR4**	Ezrin	X	✓	X	4.32	6.66	4

Accession number	Name	S	D	F	Unused Score	Coverage (%)	95% confident peptide
**sw|P52907**	F-actin-capping protein subunit alpha-1	X	✓	X	1.38	3.5	1
**tr|F8W9N7**	F-actin-capping protein subunit alpha-2	X	X	✓	0.88	16.09	2
**sw|Q01469**	Fatty acid-binding protein, epidermal	✓	✓	✓	8.27	43.7	5
**sw|Q5SYB0**	FERM and PDZ domain-containing protein 1	X	✓	X	2.49	0.51	1
**tr|C9JME2**	FERM, RhoGEF and pleckstrin domain-containing protein 1	X	✓	X	0.3	0.65	1
**sw|P02792**	Ferritin light chain	X	X	✓	2.77	12.57	2
**sw|Q9UGM5**	Fetuin-B	✓	✓	✓	6.85	11.78	6
**sw|P02671**	Fibrinogen alpha chain	✓	✓	✓	117.04	59.7	121
**sw|P02675**	Fibrinogen beta chain	✓	✓	✓	106.85	77.8	140
**sw|P02679**	Fibrinogen gamma chain	✓	✓	✓	108.8	76.38	111
**sw|Q14512**	Fibroblast growth factor-binding protein 1	X	X	✓	4.45	11.97	3
**sw|P02751**	Fibronectin	X	✓	✓	149.67	45.68	96
**sw|P23142**	Fibulin-1	✓	✓	✓	16.62	13.66	9
**sw|Q15485**	Ficolin-2	X	✓	✓	2.19	10.86	2
**sw|O75636**	Ficolin-3	✓	✓	✓	17.86	47.49	14
**sw|P20930**	Filaggrin	✓	✓	✓	48.4	21.37	31
**sw|Q5D862**	Filaggrin-2	✓	✓	X	4	0.96	2

Accession number	Name	S	D	F	Unused Score	Coverage (%)	95% confident peptide
**tr|Q60FE5**	Filamin A	X	X	✓	8.3	4.47	7
**tr|Q5HY54**	Filamin-A	X	X	✓	13.1	4.49	7
**sw|P30043**	Flavin reductase (NADPH)	X	X	✓	5.6	18.45	4
**sw|Q5H8C1**	FRAS1-related extracellular matrix protein 1	✓	X	X	4	0.78	2
**sw|P04075**	Fructose-bisphosphate aldolase A	✓	X	✓	17.88	47.8	20
**sw|Q5JSP0**	FYVE, RhoGEF and PH domain-containing protein 3	X	✓	X	2	1.24	1
**tr|G3V3R6**	Galectin	X	✓	X	4.02	14.16	3
**sw|P17931**	Galectin-3	✓	✓	✓	11.15	26.8	6
**sw|Q08380**	Galectin-3-binding protein	✓	X	✓	8.49	14.53	5
**sw|P47929**	Galectin-7	✓	✓	✓	20.92	83.09	23
**sw|Q92820**	Gamma-glutamyl hydrolase	X	X	✓	2.02	4.72	1
**sw|P17900**	Ganglioside GM2 activator	X	X	✓	2	10.36	1
**sw|P06396**	Gelsolin	✓	✓	✓	81.06	61.13	60
**tr|G3V4P8**	Glia maturation factor beta (Fragment)	X	✓	X	0.76	6	1
**tr|A0A0A0MTS2**	Glucose-6-phosphate isomerase (Fragment)	X	X	✓	8.78	14.14	6
**tr|K7ELL7**	Glucosidase 2 subunit beta	✓	✓	✓	2.04	2.43	1
**tr|A0A087X1J7**	Glutathione peroxidase	✓	✓	✓	9.04	32.44	6

Accession number	Name	S	D	F	Unused Score	Coverage (%)	95% confident peptide
**tr|H0YBE4**	Glutathione peroxidase 3 (Fragment)	✓	X	X	2.01	21.52	1
**sw|P00390**	Glutathione reductase, mitochondrial	X	✓	X	0.85	2.3	1
**sw|P78417**	Glutathione S-transferase omega-1	X	X	✓	1	3.73	1
**sw|P09211**	Glutathione S-transferase P	✓	✓	✓	11.37	35.71	7
**sw|P48637**	Glutathione synthetase	✓	X	✓	2	1.9	1
**sw|P04406**	Glyceraldehyde-3-phosphate dehydrogenase	✓	✓	✓	33.56	56.42	20
**sw|P54840**	Glycogen [starch] synthase, liver	✓	X	X	2	1.42	1
**tr|F6TLX2**	Glyoxalase domain-containing protein 4	X	X	✓	0.97	2.59	1
**sw|Q9Y625**	Glypican-6	X	✓	X	2	1.44	1
**tr|F5H0S8**	Guanine nucleotide-binding protein G(I)/G(S)/G(T) subunit beta-3 (Fragment)	✓	X	X	2	3.79	1
**sw|P00738**	Haptoglobin	✓	✓	✓	134.32	83.5	242
**sw|P00739**	Haptoglobin-related protein	✓	✓	✓	5.07	65.23	120
**tr|A0A075B6J9**	HCG1782423 (Fragment)	✓	✓	X	2	21.67	3
**tr|Q32Q12**	HCG2001850, isoform CRA_a	✓	✓	✓	4.22	15.07	3
**tr|A0A075B6K4**	HCG2043238 (Fragment)	✓	✓	X	9.68	44.44	8
**tr|A0A075B6K5**	HCG2043239 (Fragment)	✓	✓	✓	14.43	65.22	12

Accession number	Name	S	D	F	Unused Score	Coverage (%)	95% confident peptide
**sw|P08107**	Heat shock 70 kDa protein 1A/1B	✓	✓	✓	38.28	32.14	23
**sw|P34931**	Heat shock 70 kDa protein 1-like	X	✓	X	5.32	8.58	5
**sw|P11142**	Heat shock cognate 71 kDa protein	✓	✓	✓	9.33	23.22	15
**sw|P04792**	Heat shock protein beta-1	✓	✓	✓	11.14	47.32	7
**sw|P08238**	Heat shock protein HSP 90-beta	✓	✓	X	8.28	13.4	9
**sw|Q9Y5Z4**	Heme-binding protein 2	X	X	✓	2.11	13.17	3
**sw|P69905**	Hemoglobin subunit alpha	✓	✓	✓	59.98	95.07	137
**sw|P68871**	Hemoglobin subunit beta	✓	✓	✓	84.92	97.96	245
**sw|P02042**	Hemoglobin subunit delta	X	✓	✓	8.65	95.92	142
**sw|P69891**	Hemoglobin subunit gamma-1	X	X	✓	7.85	52.38	24
**sw|P69892**	Hemoglobin subunit gamma-2	X	X	✓	5.74	52.38	22
**sw|P02790**	Hemopexin	✓	✓	✓	162.5	82.25	242
**sw|P05546**	Heparin cofactor 2	✓	✓	✓	37.08	42.08	23
**sw|Q04756**	Hepatocyte growth factor activator	✓	✓	✓	8.15	14.05	7
**tr|G3XAK1**	Hepatocyte growth factor-like protein	✓	✓	✓	9.43	12.14	5
**sw|P51858**	Hepatoma-derived growth factor	✓	X	X	0.7	4.17	1
**tr|D6R9P3**	Heterogeneous nuclear ribonucleoprotein A/B	✓	X	X	2.04	3.57	1

Accession number	Name	S	D	F	Unused Score	Coverage (%)	95% confident peptide
**tr|H0YA96**	Heterogeneous nuclear ribonucleoprotein D0 (Fragment)	X	X	✓	2.2	11.43	2
**tr|A0A087WUK2**	Heterogeneous nuclear ribonucleoprotein D-like	✓	✓	X	4	4.96	2
**sw|P61978**	Heterogeneous nuclear ribonucleoprotein K	X	X	✓	2	1.94	1
**tr|S4R359**	Heterogeneous nuclear ribonucleoprotein K (Fragment)	X	✓	X	2	9	1
**sw|P22626**	Heterogeneous nuclear ribonucleoproteins A2/B1	X	✓	✓	2.22	5.95	2
**tr|G3V576**	Heterogeneous nuclear ribonucleoproteins C1/C2	X	✓	X	2	4.76	1
**sw|P26583**	High mobility group protein B2	X	X	✓	4.5	11.48	3
**tr|D6REP8**	Histidine triad nucleotide-binding protein 1	X	✓	X	0.77	17.95	1
**sw|P04196**	Histidine-rich glycoprotein	✓	✓	✓	61.26	57.33	49
**tr|U3KQK0**	Histone 1, H2bn, isoform CRA_b	✓	X	✓	8.14	35.54	21
**sw|P16403**	Histone H1.2	✓	✓	X	10	23.47	8
**sw|P16402**	Histone H1.3	X	X	✓	6.07	18.55	8
**sw|P10412**	Histone H1.4	✓	X	X	10.02	24.2	8
**sw|P16401**	Histone H1.5	✓	✓	✓	8.72	21.68	6
**tr|C9J0D1**	Histone H2A	✓	✓	X	2	7.38	1
**sw|Q93077**	Histone H2A type 1-C	X	X	✓	7.43	49.23	12
**sw|Q99878**	Histone H2A type 1-J	X	X	✓	3.91	50	14

Accession number	Name	S	D	F	Unused Score	Coverage (%)	95% confident peptide
**sw|Q6FI13**	Histone H2A type 2-A	✓	✓	✓	14	57.69	15
**sw|Q16777**	Histone H2A type 2-C	X	X	✓	12.93	62.79	15
**sw|Q9BTM1**	Histone H2A.J	✓	✓	X	12.21	49.61	14
**sw|P58876**	Histone H2B type 1-D	✓	X	X	11.78	46.83	21
**sw|O60814**	Histone H2B type 1-K	X	✓	X	7.3	46.83	21
**sw|Q99879**	Histone H2B type 1-M	✓	✓	✓	13.48	46.83	21
**sw|Q16778**	Histone H2B type 2-E	X	✓	X	1.59	46.83	18
**sw|Q8N257**	Histone H2B type 3-B	✓	X	X	4.85	35.71	11
**tr|K7EK07**	Histone H3 (Fragment)	✓	✓	✓	8.41	45.45	8
**sw|P68431**	Histone H3.1	✓	X	✓	8.23	52.94	9
**sw|Q16695**	Histone H3.1t	✓	✓	✓	5.71	27.21	8
**sw|Q71DI3**	Histone H3.2	X	X	✓	8.07	53.68	11
**tr|K7ES00**	Histone H3.3 (Fragment)	✓	X	X	9.52	23.18	8
**sw|P62805**	Histone H4	✓	✓	✓	17.4	54.37	23
**sw|Q86YZ3**	Hornerin	✓	✓	✓	9.44	6.84	7
**sw|Q14520**	Hyaluronan-binding protein 2	✓	✓	✓	11.16	14.64	7
**sw|P01876**	Ig alpha-1 chain C region	✓	✓	✓	38.38	68.27	40

Accession number	Name	S	D	F	Unused Score	Coverage (%)	95% confident peptide
**sw|P01877**	Ig alpha-2 chain C region	✓	X	✓	2.03	49.12	36
**tr|A0A087WUS7**	Ig delta chain C region	X	X	✓	1.61	6.51	2
**tr|A0A0A0MS09**	Ig delta chain C region (Fragment)	✓	✓	X	4.01	5.81	2
**tr|A0A087WV47**	Ig gamma-1 chain C region	✓	✓	✓	178.49	81.55	446
**sw|P01859**	Ig gamma-2 chain C region	✓	✓	✓	20.15	79.14	233
**tr|A0A087WXL8**	Ig gamma-3 chain C region	✓	✓	✓	42.29	68.86	241
**sw|P01861**	Ig gamma-4 chain C region	✓	✓	✓	21.49	80.43	225
**sw|P23083**	Ig heavy chain V-I region V35	✓	✓	✓	2.01	69.23	25
**sw|P01760**	Ig heavy chain V-I region W	✓	X	✓	2	18.55	26
**sw|P01773**	Ig heavy chain V-III region BUR	X	✓	X	2	24.37	11
**sw|P01767**	Ig heavy chain V-III region BUT	✓	✓	✓	2.97	56.52	31
**sw|P01781**	Ig heavy chain V-III region GAL	✓	✓	X	2.03	62.07	41
**sw|P01771**	Ig heavy chain V-III region HIL	X	X	✓	2.42	37.19	13
**sw|P01780**	Ig heavy chain V-III region J	✓	✓	X	2	32.17	11
**sw|P01780**	Ig heavy chain V-III region JON	X	✓	✓	2	32.17	11

Accession number	Name	S	D	F	Unused Score	Coverage (%)	95% confident peptide
**sw|P01762**	Ig heavy chain V-III region TR	X	✓	✓	6.97	45.08	22
**sw|P01762**	Ig heavy chain V-III region TRO	✓	X	X	6.97	45.08	22
**sw|P01779**	Ig heavy chain V-III region TUR	✓	✓	✓	7.56	50	24
**sw|P01764**	Ig heavy chain V-III region VH26	✓	X	✓	2	50.43	28
**sw|P01776**	Ig heavy chain V-III region WAS	✓	X	✓	2.05	42.74	21
**tr|A0A087X130**	Ig kappa chain C region	✓	✓	✓	23.57	67.1	122
**sw|P01593**	Ig kappa chain V-I region AG	X	X	✓	2	34.26	18
**sw|P01596**	Ig kappa chain V-I region CAR	X	✓	X	2	37.38	23
**sw|P01599**	Ig kappa chain V-I region Gal	X	✓	X	4.04	26.85	21
**sw|P01602**	Ig kappa chain V-I region HK102 (Fragment)	✓	✓	✓	4.69	49.57	29
**sw|P01604**	Ig kappa chain V-I region Kue	X	✓	X	2.01	53.7	12
**sw|P01613**	Ig kappa chain V-I region Ni	X	✓	X	4.01	33.04	10
**sw|P01608**	Ig kappa chain V-I region Roy	✓	✓	X	2	38.89	19
**sw|P01609**	Ig kappa chain V-I region Scw	✓	✓	✓	9.65	46.3	23
**sw|P01615**	Ig kappa chain V-II region FR	✓	✓	✓	2.41	25.66	3
**sw|P06310**	Ig kappa chain V-II region RPMI 6410	✓	X	X	4.14	33.08	11
**sw|P01617**	Ig kappa chain V-II region TEW	✓	✓	✓	8.25	47.79	13

Accession number	Name	S	D	F	Unused Score	Coverage (%)	95% confident peptide
**sw|P04206**	Ig kappa chain V-III region G	X	✓	X	2	59.63	28
**sw|P18136**	Ig kappa chain V-III region HIC	✓	✓	✓	19.8	78.29	49
**sw|P01621**	Ig kappa chain V-III region NG9 (Fragment)	X	X	✓	3.19	27	4
**sw|P06314**	Ig kappa chain V-IV region B17	X	X	✓	5.05	42.54	22
**sw|P06313**	Ig kappa chain V-IV region JI	✓	✓	✓	10.01	41.35	19
**sw|P06316**	Ig lambda chain V-I region BL2	✓	X	X	4.02	37.69	13
**sw|P01701**	Ig lambda chain V-I region NEW	X	X	✓	2	41.44	6
**sw|P04208**	Ig lambda chain V-I region WAH	✓	✓	✓	2	50.46	15
**sw|P01707**	Ig lambda chain V-II region TR	X	X	✓	2.01	33.33	5
**sw|P80748**	Ig lambda chain V-III region L	✓	✓	✓	8	52.25	14
**sw|P01714**	Ig lambda chain V-III region SH	✓	✓	✓	8.42	47.22	10
**tr|A0A075B6K9**	Ig lambda-2 chain C regions (Fragment)	X	X	✓	25.11	93.4	96
**sw|P0CG06**	Ig lambda-3 chain C regions	✓	X	X	21.31	93.4	96
**tr|A0A075B6L0**	Ig lambda-3 chain C regions (Fragment)	✓	✓	✓	34.26	96.23	96
**tr|A0A087WYJ9**	Ig mu chain C region	✓	✓	✓	44.68	43.91	64
**tr|A0A087WXI2**	IgGFc-binding protein	X	X	✓	8.93	4.71	8

Accession number	Name	S	D	F	Unused Score	Coverage (%)	95% confident peptide
**sw|P01591**	Immunoglobulin J chain	✓	✓	✓	21.59	46.54	24
**tr|D6RD17**	Immunoglobulin J chain (Fragment)	✓	✓	✓	4.46	38.22	21
**tr|A0A087WYR4**	Immunoglobulin lambda-like polypeptide 5	✓	✓	✓	31.67	60.43	91
**sw|O14498**	Immunoglobulin superfamily containing leucine-rich repeat protein	✓	X	X	1.89	3.04	1
**tr|A6XND0**	Insulin-like growth factor binding protein 3	X	X	✓	2.87	14.81	3
**sw|P18065**	Insulin-like growth factor-binding protein 2	✓	X	✓	2.01	2.77	1
**sw|P17936**	Insulin-like growth factor-binding protein 3	✓	✓	✓	8.05	18.21	5
**tr|H0Y5K2**	Insulin-like growth factor-binding protein 3 (Fragment)	X	✓	X	2	17.65	3
**sw|P35858**	Insulin-like growth factor-binding protein complex acid labile subunit	✓	✓	✓	30.14	32.4	15
**sw|P19827**	Inter-alpha-trypsin inhibitor heavy chain H1	✓	✓	✓	70.14	48.52	70
**sw|P19823**	Inter-alpha-trypsin inhibitor heavy chain H2	✓	✓	✓	76.8	45.14	72
**sw|Q06033**	Inter-alpha-trypsin inhibitor heavy chain H3	✓	✓	✓	20.12	33.15	29
**sw|Q14624**	Inter-alpha-trypsin inhibitor heavy chain H4	✓	✓	✓	121.77	67.53	99
**sw|P05362**	Intercellular adhesion molecule 1	X	✓	X	2	1.88	1
**tr|J3QRT5**	Intercellular adhesion molecule 2	✓	✓	X	2.07	6.37	1
**sw|Q14116**	Interleukin-18	X	✓	✓	2	4.15	1
**sw|P03956**	Interstitial collagenase	✓	✓	✓	21.74	31.98	14

Accession number	Name	S	D	F	Unused Score	Coverage (%)	95% confident peptide
**sw|P07476**	Involucrin	✓	✓	✓	26.02	29.74	18
**sw|O75874**	Isocitrate dehydrogenase [NADP] cytoplasmic	✓	X	X	2	3.14	1
**sw|Q9BWP8-10**	Isoform 10 of Collectin-11	X	✓	X	2	3.86	1
**sw|P02751-10**	Isoform 10 of Fibronectin	✓	X	X	120.32	49.54	95
**sw|P02751-13**	Isoform 13 of Fibronectin	✓	X	✓	85.8	29.42	74
**sw|Q6E0U4-16**	Isoform 16 of Dermokine	✓	✓	✓	4.01	5.81	3
**sw|P61160-2**	Isoform 2 of Actin-related protein 2	X	X	✓	1.69	3.01	1
**sw|P59998-2**	Isoform 2 of Actin-related protein 2/3 complex subunit 4	✓	X	X	2.7	5.44	3
**sw|P07355-2**	Isoform 2 of Annexin A2	✓	X	✓	13.81	34.73	10
**sw|O14791-2**	Isoform 2 of Apolipoprotein L1	✓	✓	X	10.26	19.81	7
**sw|O75882-2**	Isoform 2 of Attractin	✓	X	✓	8.77	4.95	7
**sw|P43251-2**	Isoform 2 of Biotinidase	✓	✓	✓	7.33	13.76	6
**sw|Q96IY4-2**	Isoform 2 of Carboxypeptidase B2	✓	X	X	3.11	14.44	4
**sw|P10909-2**	Isoform 2 of Clusterin	✓	✓	X	79.11	50.1	94
**sw|P81605-2**	Isoform 2 of Dermcidin	X	✓	X	4	18.18	2
**sw|P78527-2**	Isoform 2 of DNA-dependent protein kinase catalytic subunit	X	X	✓	0.88	0.32	2
**sw|P07900-2**	Isoform 2 of Heat shock protein HSP 90-alpha	✓	X	✓	17.81	14.17	10

Accession number	Name	S	D	F	Unused Score	Coverage (%)	95% confident peptide
**sw|Q9Y5Z4-2**	Isoform 2 of Heme-binding protein 2	X	✓	X	6.03	14.67	3
**sw|Q5QNW6-2**	Isoform 2 of Histone H2B type 2-F	X	✓	✓	10.98	44.03	21
**sw|P35858-2**	Isoform 2 of Insulin-like growth factor-binding protein complex acid labile subunit	X	✓	✓	15.98	30.48	15
**sw|Q06033-2**	Isoform 2 of Inter-alpha-trypsin inhibitor heavy chain H3	X	X	✓	37.69	33.3	29
**sw|P14151-2**	Isoform 2 of L-selectin	✓	✓	X	6.57	11.69	4
**sw|O00533-2**	Isoform 2 of Neural cell adhesion molecule L1-like protein	✓	X	X	4.96	2.37	3
**sw|Q86VB7-2**	Isoform 2 of Scavenger receptor cysteine-rich type 1 protein M130	X	X	✓	2.52	3.36	3
**sw|Q96P63-2**	Isoform 2 of Serpin B12	✓	X	✓	14.36	27.06	9
**sw|P04278-2**	Isoform 2 of Sex hormone-binding globulin	✓	X	X	2.14	13.54	4
**sw|P10599-2**	Isoform 2 of Thioredoxin	X	X	✓	8	38.82	4
**sw|P29401-2**	Isoform 2 of Transketolase	X	✓	X	1.68	22.03	17
**sw|P06753-2**	Isoform 2 of Tropomyosin alpha-3 chain	✓	✓	X	46.29	65.32	24
**sw|P67936-2**	Isoform 2 of Tropomyosin alpha-4 chain	✓	✓	X	6.39	59.15	23
**sw|P04070-2**	Isoform 2 of Vitamin K-dependent protein C	X	X	✓	2	7.95	3
**sw|Q02487-2**	Isoform 2B of Desmocollin-2	X	X	✓	2	1.77	1
**sw|O75882-3**	Isoform 3 of Attractin	X	✓	✓	7.38	5.01	7
**sw|P43251-3**	Isoform 3 of Biotinidase	X	X	✓	8.74	13.76	6

Accession number	Name	S	D	F	Unused Score	Coverage (%)	95% confident peptide
**sw|Q9NQ79-3**	Isoform 3 of Cartilage acidic protein 1	X	✓	X	2.12	5.57	4
**sw|P00338-3**	Isoform 3 of L-lactate dehydrogenase A chain	✓	✓	X	18.04	50.42	18
**sw|Q8IVF2-3**	Isoform 3 of Protein AHNAK2	✓	✓	X	3.46	4.04	2
**sw|P55327-3**	Isoform 3 of Tumor protein D52	X	✓	X	3.65	9.68	2
**sw|P02774-3**	Isoform 3 of Vitamin D-binding protein	✓	✓	✓	154.76	86.61	165
**sw|Q14574-2**	Isoform 3B of Desmocollin-3	X	X	✓	4.95	4.41	3
**sw|O60506-4**	Isoform 4 of Heterogeneous nuclear ribonucleoprotein Q	X	X	✓	1.33	2.47	1
**sw|P48740-4**	Isoform 4 of Mannan-binding lectin serine protease 1	X	✓	✓	8	7.15	4
**sw|Q15149-4**	Isoform 4 of Plectin	X	X	✓	0.65	2.31	11
**sw|P35030-4**	Isoform 4 of Trypsin-3	✓	✓	X	8.68	17.24	8
**sw|P10451-5**	Isoform 5 of Osteopontin	X	✓	✓	10.19	24	7
**sw|Q08499-6**	Isoform 5 of cAMP-specific 3',5'-cyclic phosphodiesterase 4D	✓	✓	✓	2.01	1.07	4
**sw|P02751-5**	Isoform 5 of Fibronectin	X	✓	X	91.34	43.06	75
**sw|Q15063-5**	Isoform 5 of Periostin	X	X	✓	19.19	21.01	11
**sw|P35241-5**	Isoform 5 of Radixin	X	X	✓	1.15	1.32	1
**sw|P06753-5**	Isoform 5 of Tropomyosin alpha-3 chain	✓	X	X	4.79	62.5	23
**sw|P02751-7**	Isoform 7 of Fibronectin	✓	X	✓	87.42	27.27	74

Accession number	Name	S	D	F	Unused Score	Coverage (%)	95% confident peptide
**sw|Q15149-7**	Isoform 7 of Plectin	X	✓	X	11.71	2.33	11
**sw|O75369-8**	Isoform 8 of Filamin-B	✓	X	X	2	0.42	1
**sw|Q15149-8**	Isoform 8 of Plectin	✓	X	X	2.02	2.32	11
**sw|P20810-9**	Isoform 9 of Calpastatin	✓	X	✓	3.33	10.67	5
**sw|P02751-9**	Isoform 9 of Fibronectin	✓	✓	X	22.93	43.13	75
**sw|Q15149-9**	Isoform 9 of Plectin	X	X	✓	3.23	2.32	11
**sw|P15924-3**	Isoform DSPIa of Desmoplakin	✓	X	✓	9.03	2.02	6
**sw|P02679-2**	Isoform Gamma-A of Fibrinogen gamma chain	X	✓	X	45.2	79.18	111
**sw|P05164-3**	Isoform H7 of Myeloperoxidase	X	X	✓	1.75	37.19	23
**sw|P13473-3**	Isoform LAMP-2C of Lysosome-associated membrane glycoprotein 2	X	✓	X	2	1.95	1
**sw|P01042-2**	Isoform LMW of Kininogen-1	✓	✓	✓	107.08	78.69	135
**sw|Q9NQ38-3**	Isoform long of Serine protease inhibitor Kazal-type 5	X	X	✓	0.81	1.19	1
**sw|Q08499-7**	Isoform N3 of cAMP-specific 3',5'-cyclic phosphodiesterase 4D	X	✓	✓	2	3.72	4
**sw|P09497-2**	Isoform Non-brain of Clathrin light chain B	X	✓	✓	6.18	12.32	3
**tr|B7ZKJ8**	ITIH4 protein	✓	✓	✓	75.41	65.99	98
**sw|P14923**	Junction plakoglobin	✓	X	✓	4.12	7.52	6
**sw|O43240**	Kallikrein-10	✓	X	✓	8.01	15.94	4

Accession number	Name	S	D	F	Unused Score	Coverage (%)	95% confident peptide
**sw|Q9UKR3**	Kallikrein-13	X	X	✓	1.6	5.78	1
**sw|Q92876**	Kallikrein-6	X	✓	✓	12.76	44.26	7
**sw|P49862**	Kallikrein-7	✓	✓	✓	7.52	16.6	4
**sw|P29622**	Kallistatin	✓	✓	✓	21.23	35.13	14
**sw|P13645**	Keratin, type I cytoskeletal 10	✓	✓	✓	82.87	63.18	144
**sw|P13646**	Keratin, type I cytoskeletal 13	✓	X	X	1.7	22.71	42
**sw|P02533**	Keratin, type I cytoskeletal 14	✓	✓	✓	71.98	64.19	61
**sw|P19012**	Keratin, type I cytoskeletal 15	X	✓	X	2.06	21.49	25
**sw|P08779**	Keratin, type I cytoskeletal 16	✓	✓	✓	86.49	72.94	79
**sw|Q04695**	Keratin, type I cytoskeletal 17	✓	✓	✓	11.62	47.22	42
**sw|P35527**	Keratin, type I cytoskeletal 9	✓	✓	✓	99.64	77.53	94
**sw|P04264**	Keratin, type II cytoskeletal 1	✓	✓	✓	116.13	70.96	210
**sw|Q7Z794**	Keratin, type II cytoskeletal 1b	X	✓	X	0.85	11.59	27
**sw|P35908**	Keratin, type II cytoskeletal 2 epidermal	✓	✓	✓	67.86	73.08	144
**sw|P19013**	Keratin, type II cytoskeletal 4	✓	X	X	1.65	14.42	21
**sw|P13647**	Keratin, type II cytoskeletal 5	✓	✓	✓	31.26	47.63	68
**sw|P02538**	Keratin, type II cytoskeletal 6A	X	✓	✓	10.8	43.09	73

Accession number	Name	S	D	F	Unused Score	Coverage (%)	95% confident peptide
**sw|P04259**	Keratin, type II cytoskeletal 6B	X	X	✓	5.17	56.56	67
**sw|P48668**	Keratin, type II cytoskeletal 6C	✓	✓	✓	77.3	56.74	73
**sw|Q8N1N4**	Keratin, type II cytoskeletal 78	X	✓	✓	2.37	8.27	21
**sw|Q5T749**	Keratinocyte proline-rich protein	X	✓	X	1.21	2.07	1
**sw|P01042**	Kininogen-1	✓	✓	✓	4.94	51.55	116
**sw|Q6YP21**	Kynurenine--oxoglutarate transaminase 3	✓	X	X	1.55	1.98	1
**tr|E7ER44**	Lactotransferrin	✓	✓	✓	58.38	45.76	55
**tr|E7EQB2**	Lactotransferrin (Fragment)	X	✓	X	7.92	46.55	55
**sw|P20700**	Lamin-B1	✓	✓	X	2	3.24	2
**sw|Q13753**	Laminin subunit gamma-2	X	✓	✓	4.03	2.18	3
**sw|P02750**	Leucine-rich alpha-2-glycoprotein	✓	✓	✓	42.28	64.27	46
**sw|P30740**	Leukocyte elastase inhibitor	✓	X	✓	4.92	13.19	4
**sw|P09960**	Leukotriene A-4 hydrolase	X	X	✓	1.36	5.07	2
**sw|P18428**	Lipopolysaccharide-binding protein	✓	✓	✓	10.65	17.46	7
**tr|G3XAP5**	L-lactate dehydrogenase	X	X	✓	2	4.98	1
**sw|P00338**	L-lactate dehydrogenase A chain	✓	✓	✓	24.82	54.82	18
**sw|P07195**	L-lactate dehydrogenase B chain	✓	✓	✓	3.85	22.46	6

Accession number	Name	S	D	F	Unused Score	Coverage (%)	95% confident peptide
**sw|P23490**	Loricrin	X	✓	X	2.01	7.69	2
**tr|M9MML0**	Low affinity immunoglobulin gamma Fc region receptor III-A	X	✓	X	4	7.24	2
**tr|M9MML6**	Low affinity immunoglobulin gamma Fc region receptor III-B	X	X	✓	2	3.72	1
**sw|P14151**	L-selectin	✓	✓	✓	6.02	12.1	4
**sw|P51884**	Lumican	✓	✓	✓	31.22	45.27	22
**sw|O95274**	Ly6/PLAUR domain-containing protein 3	✓	✓	✓	5.07	11.27	3
**sw|Q9Y5Y7**	Lymphatic vessel endothelial hyaluronic acid receptor 1	X	✓	✓	6	8.38	3
**sw|Q12912**	Lymphoid-restricted membrane protein	X	✓	X	2	1.44	2
**tr|F8VV32**	Lysozyme	X	X	✓	1.35	23.08	2
**sw|P61626**	Lysozyme C	✓	✓	✓	6.79	35.14	5
**sw|P14174**	Macrophage migration inhibitory factor	✓	✓	✓	4	17.39	3
**sw|P40121**	Macrophage-capping protein	X	X	✓	1.61	5.46	2
**tr|C9JF79**	Malate dehydrogenase (Fragment)	X	X	✓	1.36	4.05	1
**sw|P40925**	Malate dehydrogenase, cytoplasmic	✓	✓	✓	9.7	22.75	6
**sw|P40926**	Malate dehydrogenase, mitochondrial	✓	✓	✓	14.88	28.4	8
**sw|P48740**	Mannan-binding lectin serine protease 1	✓	✓	X	2.08	3.72	2
**sw|O00187**	Mannan-binding lectin serine protease 2	X	✓	✓	2.05	2.33	1

Accession number	Name	S	D	F	Unused Score	Coverage (%)	95% confident peptide
**sw|P11226**	Mannose-binding protein C	✓	✓	✓	2.01	3.63	1
**sw|P14780**	Matrix metalloproteinase-9	X	✓	✓	6.33	5.38	3
**sw|P01033**	Metalloproteinase inhibitor 1	✓	✓	✓	6.24	23.19	3
**tr|H0Y789**	Metalloproteinase inhibitor 1 (Fragment)	✓	X	X	1.05	4.24	1
**tr|E7EVA0**	Microtubule-associated protein	X	✓	X	6.08	1.79	3
**sw|P20774**	Mimecan	X	X	✓	1.94	8.73	2
**sw|P26038**	Moesin	✓	X	✓	14.76	20.28	12
**sw|P08571**	Monocyte differentiation antigen CD14	✓	✓	✓	16.51	39.73	13
**tr|F8VV13**	Mucin-like protein 1	X	✓	X	2	10.59	1
**tr|U3KPS2**	Myeloblastin	X	✓	X	1.53	4.19	1
**sw|P05164**	Myeloperoxidase	✓	✓	✓	37.91	38.79	23
**tr|B7Z6Z4**	Myosin light polypeptide 6	✓	✓	✓	10.03	26.05	5
**sw|P35579**	Myosin-9	X	X	✓	4.95	2.65	4
**tr|A0A087WZH7**	Myristoylated alanine-rich C-kinase substrate	✓	✓	✓	2.15	5.76	1
**sw|Q86U44**	N6-adenosine-methyltransferase 70 kDa subunit	✓	X	X	1.59	1.38	1
**sw|Q96PD5**	N-acetylmuramoyl-L-alanine amidase	✓	✓	✓	34.74	47.92	23
**tr|H7C2G2**	NAD(P)(+)--arginine ADP-ribosyltransferase (Fragment)	X	X	✓	0.68	2.56	1

Accession number	Name	S	D	F	Unused Score	Coverage (%)	95% confident peptide
**sw|E9PAV3**	Nascent polypeptide-associated complex subunit alpha, muscle-specific form	✓	✓	✓	4	1.4	2
**tr|A0A087WX77**	Neural cell adhesion molecule 1	✓	✓	X	6	3.73	3
**sw|O00533**	Neural cell adhesion molecule L1-like protein	X	✓	X	2.02	2.4	3
**sw|Q09666**	Neuroblast differentiation-associated protein AHNAK	✓	✓	✓	24.03	12.99	19
**sw|P22894**	Neutrophil collagenase	X	X	✓	2.01	3.43	1
**sw|P59666**	Neutrophil defensin 3	✓	✓	✓	5.83	20.21	3
**sw|P08246**	Neutrophil elastase	✓	✓	✓	1.18	6.37	2
**tr|X6R8F3**	Neutrophil gelatinase-associated lipocalin	✓	✓	✓	8.17	44	6
**sw|P43490**	Nicotinamide phosphoribosyltransferase	X	X	✓	1.18	6.11	2
**sw|Q99784**	Noelin	✓	X	X	1.19	2.27	1
**sw|P05204**	Non-histone chromosomal protein HMG-17	✓	X	X	1.28	8.89	1
**sw|P10153**	Non-secretory ribonuclease	X	X	✓	2	9.32	1
**tr|H3BRV9**	Nuclear transport factor 2 (Fragment)	✓	X	X	0.67	7.48	1
**sw|P19338**	Nucleolin	✓	✓	✓	7.03	4.79	4
**sw|P06748**	Nucleophosmin	✓	✓	✓	4.44	7.48	3
**sw|P10451**	Osteopontin	X	X	✓	1.07	19.11	6

Accession number	Name	S	D	F	Unused Score	Coverage (%)	95% confident peptide
**sw|Q6S8J3**	POTE ankyrin domain family member E	X	✓	X	2	8.09	17
**sw|P00995**	Pancreatic secretory trypsin inhibitor	X	X	✓	1.85	10.13	1
**sw|O95497**	Pantetheinase	X	✓	✓	2.04	1.75	1
**tr|F5GXR3**	Parathymosin	X	X	✓	1.31	10.58	1
**sw|P26022**	Pentraxin-related protein PTX3	✓	X	X	1.54	2.62	1
**sw|Q6UXB8**	Peptidase inhibitor 16	✓	✓	✓	13.18	20.95	9
**tr|F8WE65**	Peptidyl-prolyl cis-trans isomerase	✓	X	X	2	34.17	4
**sw|P62937**	Peptidyl-prolyl cis-trans isomerase A	✓	✓	✓	16.87	50.3	11
**sw|P23284**	Peptidyl-prolyl cis-trans isomerase B	✓	X	✓	6	16.2	3
**sw|Q00688**	Peptidyl-prolyl cis-trans isomerase FKBP3	✓	X	X	1.72	3.57	1
**sw|O60664**	Perilipin-3	✓	✓	X	7.44	11.75	4
**sw|Q06830**	Peroxiredoxin-1	✓	✓	✓	22.13	55.78	15
**tr|A0A0A0MSI0**	Peroxiredoxin-1 (Fragment)	✓	✓	✓	7.44	52.05	14
**sw|P32119**	Peroxiredoxin-2	✓	✓	✓	12.21	42.42	11
**sw|P30041**	Peroxiredoxin-6	X	X	✓	2	4.46	1
**tr|H0YCQ3**	Phosphatidate phosphatase PPAPDC1B (Fragment)	✓	X	X	2	2.86	1
**sw|P04180**	Phosphatidylcholine-sterol acyltransferase	✓	✓	✓	4.31	13.86	4

Accession number	Name	S	D	F	Unused Score	Coverage (%)	95% confident peptide
**sw|P30086**	Phosphatidylethanolamine-binding protein 1	✓	✓	✓	6.09	20.32	3
**sw|P80108**	Phosphatidylinositol-glycan-specific phospholipase D	✓	✓	✓	10.11	7.14	5
**sw|P00558**	Phosphoglycerate kinase 1	✓	✓	✓	16.74	28.78	9
**sw|P18669**	Phosphoglycerate mutase 1	✓	✓	✓	7.47	24.8	4
**sw|P15259**	Phosphoglycerate mutase 2	X	✓	X	3.17	9.88	2
**sw|P55058**	Phospholipid transfer protein	✓	✓	✓	5.62	7.71	3
**sw|P36955**	Pigment epithelium-derived factor	✓	✓	✓	32.46	49.76	24
**sw|P03952**	Plasma kallikrein	✓	✓	✓	40.7	43.89	30
**tr|H0YAC1**	Plasma kallikrein (Fragment)	✓	✓	✓	51.25	40.82	30
**sw|P05155**	Plasma protease C1 inhibitor	✓	✓	✓	59.58	53.8	64
**sw|P05154**	Plasma serine protease inhibitor	✓	✓	✓	3.87	9.61	5
**sw|P00747**	Plasminogen	✓	✓	✓	109	75.43	71
**sw|P13796**	Plastin-2	✓	✓	✓	32.79	41.79	18
**sw|P02775**	Platelet basic protein	X	✓	✓	3.96	18.75	2
**sw|P10720**	Platelet factor 4 variant	X	X	✓	4	23.08	2
**tr|A0A0C4DG49**	Poliovirus receptor	✓	✓	X	5.59	7.67	3
**tr|Q96C32**	Polyubiquitin-C	X	X	✓	2.74	44.59	4

Accession number	Name	S	D	F	Unused Score	Coverage (%)	95% confident peptide
**sw|P20742**	Pregnancy zone protein	✓	✓	✓	6.01	17.07	76
**sw|P02545**	Prelamin-A/C	✓	✓	✓	25.05	27.26	16
**sw|Q8N0Y7**	Probable phosphoglycerate mutase 4	X	X	✓	2	5.51	1
**sw|P07737**	Profilin-1	✓	✓	✓	9.92	60	6
**sw|P12273**	Prolactin-inducible protein	X	✓	X	2	8.22	1
**sw|Q9ULL5**	Proline-rich protein 12	X	✓	X	2	0.66	1
**sw|P27918**	Properdin	✓	✓	✓	17.71	24.09	12
**tr|C9JIZ6**	Prosaposin	X	X	✓	2.86	3.23	2
**tr|A6XMV9**	Protease serine 2 preproprotein	✓	✓	X	9.74	24.14	43
**tr|G3V5Z7**	Proteasome subunit alpha type	X	✓	X	1.32	4.76	1
**sw|P25788**	Proteasome subunit alpha type-3	✓	X	X	2.11	4.71	1
**tr|H0YN18**	Proteasome subunit alpha type-4	✓	X	X	2	3.48	1
**sw|P28066**	Proteasome subunit alpha type-5	✓	✓	X	6.32	17.43	4
**sw|O14818**	Proteasome subunit alpha type-7	✓	X	✓	2.63	8.87	2
**sw|P20618**	Proteasome subunit beta type-1	X	X	✓	2.09	5.81	1
**sw|P28074**	Proteasome subunit beta type-5	✓	X	✓	6	12.93	3
**sw|P28072**	Proteasome subunit beta type-6	✓	✓	✓	8	17.57	4

Accession number	Name	S	D	F	Unused Score	Coverage (%)	95% confident peptide
**sw|P28065**	Proteasome subunit beta type-9	✓	X	X	2.02	4.57	1
**sw|Q8IVF2**	Protein AHNAK2	X	X	✓	2.01	3.97	2
**sw|P02760**	Protein AMBP	✓	✓	✓	37.5	53.41	39
**tr|K7ER74**	Protein AP	✓	✓	✓	10	40.45	7
**tr|K7ER74**	Protein APOC4-APOC2	✓	✓	✓	10	40.45	7
**tr|F8WCF6**	Protein ARPC4-TTLL3	X	✓	✓	4.61	18.78	3
**tr|K7ELW0**	Protein deglycase DJ-1	✓	X	X	0.52	8.28	2
**sw|P07237**	Protein disulfide-isomerase	✓	✓	✓	7.84	17.13	5
**sw|P30101**	Protein disulfide-isomerase A3	X	X	✓	5.42	11.49	5
**sw|P13667**	Protein disulfide-isomerase A4	X	X	✓	0.87	2.79	2
**tr|A0A0C4DH31**	Protein IGHV1-18 (Fragment)	✓	✓	X	16	55.56	30
**tr|A0A0C4DH33**	Protein IGHV1-24 (Fragment)	✓	✓	X	2	57.26	15
**tr|A0A0B4J1V4**	Protein IGHV1-46 (Fragment)	✓	✓	✓	3.62	51.28	30
**tr|A0A0B4J1V5**	Protein IGHV1-69 (Fragment)	X	✓	X	2	59.83	30
**tr|A0A0B4J2H0**	Protein IGHV1-69-2 (Fragment)	✓	✓	✓	4.08	59.83	30
**tr|A0A0B4J1V2**	Protein IGHV2-26 (Fragment)	X	✓	X	2.05	11.76	2

Accession number	Name	S	D	F	Unused Score	Coverage (%)	95% confident peptide
**tr|A0A0C4DH43**	Protein IGHV2-70 (Fragment)	✓	X	X	2	12.61	3
**tr|A0A075B7D8**	Protein IGHV3	X	✓	X	0.56	42.02	29
**tr|A0A0B4J1V0**	Protein IGHV3-15 (Fragment)	✓	✓	✓	9.1	78.99	31
**tr|A0A0B4J2B7**	Protein IGHV3-30 (Fragment)	✓	✓	✓	14.25	64.1	44
**tr|A0A0B4J1V3**	Protein IGHV3-33 (Fragment)	X	✓	X	2.68	64.1	48
**tr|A0A0B4J1X8**	Protein IGHV3-43 (Fragment)	✓	X	✓	4.02	54.24	29
**tr|A0A0A0MS15**	Protein IGHV3-49 (Fragment)	✓	✓	✓	13.34	62.18	19
**tr|A0A087WSX4**	Protein IGHV3-53 (Fragment)	X	✓	✓	2	41.38	21
**tr|A0A0B4J1V6**	Protein IGHV3-73 (Fragment)	✓	X	✓	6.15	73.11	18
**tr|A0A0B4J1X5**	Protein IGHV3-74 (Fragment)	✓	✓	✓	4.39	72.65	60
**tr|A0A0C4DH34**	Protein IGHV4-28 (Fragment)	✓	✓	X	1.71	44.44	11
**tr|A0A087WSY4**	Protein IGHV4-31 (Fragment)	X	✓	X	2	19.49	10
**tr|A0A0A0MS12**	Protein IGHV4-34 (Fragment)	X	✓	X	8.07	33.33	16

Accession number	Name	S	D	F	Unused Score	Coverage (%)	95% confident peptide
**tr|A0A0C4DH41**	Protein IGHV4-61 (Fragment)	✓	✓	✓	14.29	50.85	18
**tr|A0A0C4DH38**	Protein IGHV5-51 (Fragment)	✓	✓	✓	9.51	52.99	30
**tr|A0A0A0MT74**	Protein IGKV1-16 (Fragment)	✓	✓	X	2.7	49.57	19
**tr|A0A0B4J1Z4**	Protein IGKV1-17 (Fragment)	✓	✓	✓	8.16	52.14	32
**tr|A0A087WVN6**	Protein IGKV1-27	X	✓	✓	6.03	47.42	34
**tr|A0A075B6S5**	Protein IGKV1-27 (Fragment)	X	X	✓	4.12	39.32	34
**tr|A0A0B4J1U0**	Protein IGKV1-33 (Fragment)	✓	✓	✓	8	39.32	19
**tr|A0A0B4J1Z7**	Protein IGKV1-39 (Fragment)	✓	✓	X	10	49.57	39
**tr|A0A0C4DH72**	Protein IGKV1-6 (Fragment)	✓	X	X	2	47.86	23
**tr|A0A0C4DH69**	Protein IGKV1-9 (Fragment)	X	X	✓	2	49.57	19
**tr|A0A0B4J1Z3**	Protein IGKV1D-16 (Fragment)	X	X	✓	1.34	41.03	29
**tr|A0A0C4DH68**	Protein IGKV2-24 (Fragment)	✓	X	X	0.34	38.33	5
**tr|A0A087X1V9**	Protein IGKV2-28	X	✓	✓	6	46.08	13

Accession number	Name	S	D	F	Unused Score	Coverage (%)	95% confident peptide
**tr|A0A087X0Q4**	Protein IGKV2-40	✓	✓	X	6.74	59.62	13
**tr|A0A075B6R9**	Protein IGKV2D-24 (Fragment)	✓	✓	X	6.01	45.83	6
**tr|A0A087X0P6**	Protein IGKV2D-29	✓	✓	✓	2.1	39.22	9
**tr|A0A075B6S6**	Protein IGKV2D-30 (Fragment)	X	✓	X	0.64	36.67	11
**tr|A0A087WZW8**	Protein IGKV3-11	✓	✓	✓	39.13	76.39	122
**tr|A0A0B4J1T9**	Protein IGKV3-15 (Fragment)	✓	X	X	8	47.83	29
**tr|A0A0B4J1Z6**	Protein IGKV3-20 (Fragment)	X	✓	✓	8.02	75.86	47
**tr|A0A087WSY6**	Protein IGKV3D-15 (Fragment)	X	✓	✓	7.77	47.83	19
**tr|A0A0C4DH24**	Protein IGKV6-21 (Fragment)	✓	X	✓	4	42.98	4
**tr|A0A0A0MT36**	Protein IGKV6D-21 (Fragment)	✓	✓	✓	4.39	37.72	3
**tr|A0A075B6I4**	Protein IGLV10-54 (Fragment)	✓	✓	X	4	17.95	2
**tr|A0A075B6J0**	Protein IGLV1-40 (Fragment)	✓	✓	✓	5.94	53.39	12
**tr|A0A0B4J1U1**	Protein IGLV1-44 (Fragment)	✓	✓	X	3.64	52.99	12

Accession number	Name	S	D	F	Unused Score	Coverage (%)	95% confident peptide
**tr|A0A075B6I8**	Protein IGLV1-47 (Fragment)	✓	✓	✓	11.19	52.14	16
**tr|A0A075B6I6**	Protein IGLV1-50 (Fragment)	X	X	✓	1.82	41.53	8
**tr|A0A075B6I5**	Protein IGLV1-51 (Fragment)	✓	✓	✓	6	51.24	14
**tr|A0A075B6K3**	Protein IGLV2-11 (Fragment)	✓	✓	✓	2.16	39.5	8
**tr|A0A075B6K1**	Protein IGLV2-14 (Fragment)	✓	✓	✓	2	32.5	7
**tr|A0A075B6J8**	Protein IGLV3-19 (Fragment)	✓	✓	✓	7.26	52.68	9
**tr|A0A075B6J7**	Protein IGLV3-21 (Fragment)	X	✓	✓	6.38	52.14	9
**tr|A0A075B6J4**	Protein IGLV3-25 (Fragment)	✓	✓	✓	9.7	53.57	10
**tr|A0A075B6J3**	Protein IGLV3-27 (Fragment)	✓	✓	✓	4	28.32	7
**tr|A0A075B6H9**	Protein IGLV4-69 (Fragment)	✓	✓	X	3.51	37.82	3
**tr|A0A087WSX0**	Protein IGLV5-45 (Fragment)	✓	✓	X	2	13.82	1
**tr|A0A0B4J1U2**	Protein IGLV7-43 (Fragment)	✓	✓	✓	3.69	21.37	3
**tr|A0A075B6I9**	Protein IGLV7-46 (Fragment)	✓	✓	X	4.92	21.55	3
**tr|A0A075B6I0**	Protein IGLV8-61 (Fragment)	✓	✓	✓	4	20.49	4
**tr|A0A0B4J1Y8**	Protein IGLV9-49	✓	✓	✓	6.17	24.39	3

Accession number	Name	S	D	F	Unused Score	Coverage (%)	95% confident peptide
**sw|Q9BUN1**	Protein MENT	✓	X	X	1.48	4.4	2
**sw|Q92597**	Protein NDRG1	X	X	✓	2.01	3.55	1
**tr|G5E9F8**	Protein S (Alpha), isoform CRA_b	✓	X	X	0.35	8.99	4
**tr|R4GN98**	Protein S100 (Fragment)	✓	✓	✓	2	9.41	2
**sw|P31949**	Protein S100-A11	✓	✓	✓	4.88	32.38	3
**sw|Q9HCY8**	Protein S100-A14	✓	X	X	2	6.73	1
**sw|Q96FQ6**	Protein S100-A16	X	✓	X	2.99	22.33	2
**sw|P29034**	Protein S100-A2	X	✓	✓	6.65	44.9	4
**sw|P26447**	Protein S100-A4	X	X	✓	1.74	19.8	2
**sw|P06703**	Protein S100-A6	X	✓	X	2.01	8.89	2
**sw|P31151**	Protein S100-A7	✓	X	✓	6.57	34.65	4
**sw|Q86SG5**	Protein S100-A7A	X	✓	✓	2	10.89	1
**sw|P05109**	Protein S100-A8	✓	✓	✓	19.18	75.27	22
**sw|P06702**	Protein S100-A9	✓	✓	✓	21.49	78.95	27
**sw|P25815**	Protein S100-P	X	✓	✓	4	24.21	2
**tr|A0A096LPE2**	Protein SAA2-SAA4	✓	✓	✓	18.78	46.15	17
**tr|A0A0C4DFV9**	Protein SET	✓	✓	X	2	3.76	1

Accession number	Name	S	D	F	Unused Score	Coverage (%)	95% confident peptide
**sw|Q8TF72**	Protein Shroom3	✓	✓	X	2.45	0.45	2
**tr|A0A075B6Z2**	Protein TRAJ56 (Fragment)	✓	✓	✓	2	38.1	4
**tr|G3V2W1**	Protein Z-dependent protease inhibitor	✓	X	✓	6.14	10.12	4
**sw|Q08188**	Protein-glutamine gamma-glutamyltransferase E	✓	X	X	1.45	2.74	2
**sw|P00734**	Prothrombin	✓	✓	✓	79.9	65.76	70
**tr|B8ZZQ6**	Prothymosin alpha	✓	✓	X	4.1	22.43	3
**sw|P00491**	Purine nucleoside phosphorylase	X	X	✓	5.24	14.88	3
**sw|A6NMY6**	Putative annexin A2-like protein	X	X	✓	7.19	10.91	4
**sw|Q5VTE0**	Putative elongation factor 1-alpha-like 3	✓	✓	✓	22.02	28.57	14
**sw|Q5VSP4**	Putative lipocalin 1-like protein 1	✓	X	X	2	6.79	2
**sw|Q8NFI4**	Putative protein FAM10A5	✓	X	X	1.17	1.9	1
**tr|A0A096LP77**	Putative trypsin-6	X	X	✓	22	24.14	43
**tr|H3BTN5**	Pyruvate kinase (Fragment)	X	✓	X	2.78	36.7	13
**sw|P14618**	Pyruvate kinase PKM	✓	✓	✓	28.76	39.36	15
**sw|P50395**	Rab GDP dissociation inhibitor beta	X	X	✓	5.09	10.79	3
**tr|F6WQW2**	Ran-specific GTPase-activating protein	X	✓	X	0.97	3.24	1
**sw|P46940**	Ras GTPase-activating-like protein IQGAP1	X	X	✓	2.72	1.99	2

Accession number	Name	S	D	F	Unused Score	Coverage (%)	95% confident peptide
**sw|P61026**	Ras-related protein Rab-10	✓	X	X	2.08	11.5	2
**tr|C9J8S2**	Retinoic acid receptor responder protein 2 (Fragment)	✓	✓	✓	3.66	13.84	2
**tr|Q5VY30**	Retinol binding protein 4, plasma, isoform CRA_b	✓	✓	✓	20.03	73.37	19
**tr|A0A0C4DGV7**	Retinol-binding protein 4	X	X	✓	4.69	73.37	19
**sw|A6NKG5**	Retrotransposon-like protein 1	✓	X	X	2	0.44	1
**sw|Q53RT3**	Retroviral-like aspartic protease 1	✓	✓	X	4	7.58	2
**sw|P52566**	Rho GDP-dissociation inhibitor 2	✓	X	X	1.55	10.45	2
**tr|H0YGX7**	Rho GDP-dissociation inhibitor 2 (Fragment)	X	✓	✓	3.59	10.77	2
**sw|Q96PE2**	Rho guanine nucleotide exchange factor 17	✓	X	X	2	0.39	2
**sw|Q8N443**	RIB43A-like with coiled-coils protein 1	X	✓	X	2	1.85	1
**sw|P34096**	Ribonuclease 4	✓	X	✓	4.13	16.33	2
**sw|P13489**	Ribonuclease inhibitor	✓	X	✓	9.72	14.1	5
**tr|C9JHR8**	Scavenger receptor cysteine-rich type 1 protein M130	✓	X	X	1.89	3.38	3
**tr|Q5K684**	SCCA1/SCCA2 fusion protein	X	X	✓	4.26	9.74	3
**sw|Q13103**	Secreted phosphoprotein 24	✓	✓	X	2.03	5.69	1
**sw|P49908**	Selenoprotein P	X	✓	X	4.18	10.24	4
**tr|D6REX5**	Selenoprotein P (Fragment)	✓	✓	✓	6.29	12.58	4

Accession number	Name	S	D	F	Unused Score	Coverage (%)	95% confident peptide
**sw|Q16181**	Septin-7	✓	X	X	0.95	2.06	1
**tr|I3L3D7**	Serine protease 33 (Fragment)	✓	X	X	0.58	2.97	1
**sw|Q9H0K1**	Serine/threonine-protein kinase SIK2	X	X	✓	0.62	0.97	1
**sw|P02787**	Serotransferrin	✓	✓	✓	256.5	87.68	586
**sw|Q8IW75**	Serpin A12	X	✓	✓	2.09	2.17	3
**tr|H0Y5H9**	Serpin B4 (Fragment)	X	✓	X	2.05	10.24	3
**sw|P36952**	Serpin B5	✓	✓	✓	7.07	13.87	5
**sw|P02768**	Serum albumin	✓	✓	✓	386.02	93.6	930
**tr|E9PQD6**	Serum amyloid A protein	X	✓	X	2.94	42.62	4
**sw|P0DJI8**	Serum amyloid A-1 protein	✓	✓	✓	17.4	66.39	13
**sw|P0DJI9**	Serum amyloid A-2 protein	X	✓	✓	6	64.75	10
**sw|P02743**	Serum amyloid P-component	✓	✓	✓	16.17	33.18	10
**sw|P27169**	Serum paraoxonase/arylesterase 1	✓	✓	✓	24.84	57.46	22
**tr|B0FWH6**	Sex hormone binding globulin (Fragment)	X	X	✓	2	10.68	1
**sw|P04278**	Sex hormone-binding globulin	✓	✓	✓	9.46	17.91	5
**tr|A0A0C4DGN2**	Sex hormone-binding globulin (Fragment)	✓	X	X	4.52	15	4
**tr|A0A087WV2**	SH3 domain-binding glutamic acid-rich-like protein 3	✓	✓	✓	2	4.43	1

Accession number	Name	S	D	F	Unused Score	Coverage (%)	95% confident peptide
**3**							
**sw|A6NMB1**	Sialic acid-binding Ig-like lectin 16	✓	✓	X	0.66	1.46	1
**tr|E7EUY5**	Single-stranded DNA-binding protein, mitochondrial (Fragment)	✓	✓	✓	2	5.93	1
**sw|Q5T750**	Skin-specific protein 32	✓	✓	✓	2	3.2	1
**sw|P62318**	Small nuclear ribonucleoprotein Sm D3	✓	X	✓	2	7.14	1
**tr|A8MUA9**	SMT3 suppressor of mif two 3 homolog 3 (Yeast), isoform CRA_d	✓	✓	✓	2	8.89	1
**sw|Q9Y5Y9**	Sodium channel protein type 10 subunit alpha	✓	X	X	2	0.41	1
**tr|M0QZQ3**	Spectrin beta chain, non-erythrocytic 4	✓	X	X	2.54	0.85	2
**sw|Q9P0W8**	Spermatogenesis-associated protein 7	X	X	✓	1.13	1.17	1
**sw|P08254**	Stromelysin-1	X	X	✓	14.08	18.24	9
**sw|O00391**	Sulfhydryl oxidase 1	X	X	✓	1.49	2.28	2
**sw|P00441**	Superoxide dismutase [Cu-Zn]	✓	✓	✓	2.14	9.09	1
**sw|Q6UWP8**	Suprabasin	✓	✓	✓	40.65	64.92	23
**sw|P18827**	Syndecan-1	X	X	✓	2	5.48	1
**tr|H7C1K4**	Syndecan-1 (Fragment)	✓	X	X	2	7.91	1
**sw|Q9Y490**	Talin-1	X	X	✓	4.68	2.13	3
**sw|Q5TCY1**	Tau-tubulin kinase 1	✓	X	✓	2	0.61	3

Accession number	Name	S	D	F	Unused Score	Coverage (%)	95% confident peptide
**tr|E9PCA1**	T-complex protein 1 subunit epsilon	✓	X	X	2.39	3.27	2
**sw|Q99832**	T-complex protein 1 subunit eta	X	X	✓	3.58	3.13	2
**tr|D6RFY8**	T-complex protein 11 homolog	✓	✓	X	2	14.86	2
**tr|A0A087X0I0**	Tenascin-X	X	✓	X	1.19	0.66	1
**sw|P05452**	Tetranectin	✓	✓	✓	24	68.32	18
**tr|G8JLH6**	Tetraspanin (Fragment)	X	X	✓	2	4.39	1
**sw|P10599**	Thioredoxin	✓	✓	✓	9.23	42.86	6
**tr|I3L2R6**	Thioredoxin domain-containing protein 17	X	✓	X	0.61	9.18	1
**sw|P07996**	Thrombospondin-1	X	✓	✓	10.31	5.98	5
**sw|P35443**	Thrombospondin-4	✓	✓	✓	2	1.04	1
**sw|P19971**	Thymidine phosphorylase	X	X	✓	2	3.11	1
**sw|P05543**	Thyroxine-binding globulin	✓	✓	✓	25.5	36.87	16
**tr|A0A0A0MRA3**	Titin	✓	✓	✓	2.01	0.08	8
**sw|P37837**	Transaldolase	✓	✓	✓	6.02	18.99	4
**tr|A0A024RAC6**	Transcription elongation factor B (SIII), polypeptide 3 (110kDa, elongin A), isoform CRA_a	✓	X	X	1.51	1.04	2
**sw|Q6VUC0**	Transcription factor AP-2-epsilon	✓	✓	X	2	1.58	3

Accession number	Name	S	D	F	Unused Score	Coverage (%)	95% confident peptide
**tr|A0A087X0R4**	Transducin-like enhancer protein 6	✓	X	X	1.37	3.13	1
**sw|P02786**	Transferrin receptor protein 1	✓	X	X	1.38	1.71	1
**sw|Q15582**	Transforming growth factor-beta-induced protein ig-h3	✓	X	✓	27.68	37.04	20
**tr|H0Y8L3**	Transforming growth factor-beta-induced protein ig-h3 (Fragment)	✓	X	X	0.42	4.65	2
**tr|X6RJP6**	Transgelin-2 (Fragment)	✓	✓	✓	10	29.41	6
**sw|P55072**	Transitional endoplasmic reticulum ATPase	✓	✓	✓	12	10.92	6
**sw|P29401**	Transketolase	✓	✓	✓	27.54	22.31	17
**tr|Q5W0H4**	Translationally-controlled tumor protein	✓	X	✓	6	18.09	4
**sw|Q66K66**	Transmembrane protein 198	✓	X	X	2.01	2.5	4
**tr|C9JXI5**	Transmembrane protein 198 (Fragment)	✓	✓	✓	2	3.97	4
**sw|P02766**	Transthyretin	✓	✓	✓	34.3	79.59	51
**sw|P60174**	Triosephosphate isomerase	✓	✓	✓	27.11	55.59	15
**sw|Q14134**	Tripartite motif-containing protein 29	✓	X	X	3.82	3.4	2
**tr|H0Y4A2**	tRNA (cytosine(38)-C(5))-methyltransferase (Fragment)	X	✓	X	2	8.14	1
**tr|A0A087WWU8**	Tropomyosin alpha-3 chain	X	✓	X	1.51	65.64	23
**sw|P67936**	Tropomyosin alpha-4 chain	✓	✓	✓	40.2	65.73	25

Accession number	Name	S	D	F	Unused Score	Coverage (%)	95% confident peptide
**tr|E7EQ64**	Trypsin-1	✓	✓	✓	2	22.22	38
**sw|P35030**	Trypsin-3	X	X	✓	4.16	12.83	7
**sw|Q71U36**	Tubulin alpha-1A chain	✓	✓	X	19.41	30.16	11
**tr|F5H5D3**	Tubulin alpha-1C chain	✓	✓	✓	7.82	26.2	11
**sw|Q6PEY2**	Tubulin alpha-3E chain	X	X	✓	1.77	3.56	1
**sw|P07437**	Tubulin beta chain	✓	✓	X	1.28	31.53	13
**sw|P68371**	Tubulin beta-4B chain	✓	X	X	17.87	28.09	11
**sw|Q3ZCM7**	Tubulin beta-8 chain	X	✓	X	2.19	6.53	2
**sw|Q9BW30**	Tubulin polymerization-promoting protein family member 3	✓	X	X	6.63	21.59	4
**tr|E5RJD8**	Tubulin-specific chaperone A	X	✓	X	0.56	8.4	1
**sw|B5MCY1**	Tudor domain-containing protein 15	X	✓	X	1.38	0.41	1
**sw|Q9NV31**	U3 small nucleolar ribonucleoprotein protein IMP3	X	✓	✓	2	3.8	1
**sw|P62310**	U6 snRNA-associated Sm-like protein LSm3	X	✓	✓	2.02	11.76	1
**sw|O95777**	U6 snRNA-associated Sm-like protein LSm8	X	✓	✓	2	10.42	1
**tr|J3KR44**	Ubiquitin thioesterase	✓	X	X	0.61	3.31	1
**tr|J3QTR3**	Ubiquitin-40S ribosomal protein S27a (Fragment)	X	X	✓	6.01	32.08	4
**sw|P62987**	Ubiquitin-60S ribosomal protein L40	✓	✓	✓	6	26.56	4

Accession number	Name	S	D	F	Unused Score	Coverage (%)	95% confident peptide
**sw|P68036**	Ubiquitin-conjugating enzyme E2 L3	X	X	✓	1.7	5.84	1
**tr|Q5QPP4**	UDP-glucose 4-epimerase (Fragment)	✓	✓	X	2	3.35	1
**tr|B4E1Z4**	Uncharacterized protein	✓	✓	✓	146.28	51.03	105
**sw|Q68DL7**	Uncharacterized protein C18orf63	✓	✓	✓	2	1.02	1
**tr|G3V394**	Unconventional myosin-Va	✓	✓	X	0.72	0.54	1
**tr|F5GXR6**	Unconventional myosin-XVIIIb	✓	X	X	0.47	0.43	1
**sw|P54727**	UV excision repair protein RAD23 homolog B	✓	✓	X	4.09	9.29	2
**sw|P19320**	Vascular cell adhesion protein 1	✓	✓	✓	12	8.39	6
**sw|Q6EMK4**	Vasorin	✓	✓	X	4	3.27	2
**tr|D6RGZ6**	Versican core protein (Fragment)	✓	X	X	4.72	3.95	3
**sw|P08670**	Vimentin	✓	✓	✓	67.04	56.01	44
**sw|P18206**	Vinculin	X	X	✓	4.29	3.79	3
**sw|P02774**	Vitamin D-binding protein	✓	✓	✓	79.36	90.08	165
**tr|E7END6**	Vitamin K-dependent protein C	✓	X	X	4.23	8.28	3
**sw|P07225**	Vitamin K-dependent protein S	✓	✓	✓	20.17	13.31	12
**sw|P04004**	Vitronectin	✓	✓	✓	43.99	61.3	45
**sw|P04275**	von Willebrand factor	✓	✓	✓	13.67	6.43	12

Accession number	Name	S	D	F	Unused Score	Coverage (%)	95% confident peptide
**sw|O75083**	WD repeat-containing protein 1	✓	✓	X	1.78	2.31	1
**sw|P12955**	Xaa-Pro dipeptidase	✓	X	X	2	2.03	1
**sw|Q9NQZ6**	Zinc finger C4H2 domain-containing protein	X	X	✓	2	3.13	2
**sw|P25311**	Zinc-alpha-2-glycoprotein	✓	✓	✓	69.18	69.8	73
